# Structural Insights into the SPRED1-Neurofibromin-KRAS Complex and Disruption of SPRED1-Neurofibromin Interaction by Oncogenic EGFR

**DOI:** 10.1016/j.celrep.2020.107909

**Published:** 2020-07-21

**Authors:** Wupeng Yan, Evan Markegard, Srisathiyanarayanan Dharmaiah, Anatoly Urisman, Matthew Drew, Dominic Esposito, Klaus Scheffzek, Dwight V. Nissley, Frank McCormick, Dhirendra K. Simanshu

**Affiliations:** 1 NCI RAS Initiative, Cancer Research Technology Program, Frederick National Laboratory for Cancer Research, Leidos Biomedical Research, Inc., Frederick, MD 21701, USA; 2 Helen Diller Family Comprehensive Cancer Center, University of California, San Francisco, San Francisco, CA 94158, USA; 3 Institute of Biological Chemistry, Biocenter, Medical University of Innsbruck, 6020 Innsbruck, Austria; 4 These authors contributed equally; 5 Lead Contact

## Abstract

Sprouty-related, EVH1 domain-containing (SPRED) proteins negatively regulate RAS/mitogen-activated protein kinase (MAPK) signaling following growth factor stimulation. This inhibition of RAS is thought to occur primarily through SPRED1 binding and recruitment of neurofibromin, a RasGAP, to the plasma membrane. Here, we report the structure of neurofibromin (GTPase-activating protein [GAP]-related domain) complexed with SPRED1 (EVH1 domain) and KRAS. The structure provides insight into how the membrane targeting of neurofibromin by SPRED1 allows simultaneous interaction with activated KRAS. *SPRED1* and *NF1* loss-of-function mutations occur across multiple cancer types and developmental diseases. Analysis of the neurofibromin-SPRED1 interface provides a rationale for mutations observed in Legius syndrome and suggests why SPRED1 can bind to neurofibromin but no other RasGAPs. We show that oncogenic EGFR(L858R) signaling leads to the phosphorylation of SPRED1 on serine 105, disrupting the SPRED1-neurofibromin complex. The structural, biochemical, and biological results provide new mechanistic insights about how SPRED1 interacts with neurofibromin and regulates active KRAS levels in normal and pathologic conditions.

## In Brief

Yan et al. describe the structure of neurofibromin (GAP-related domain) in complex with SPRED1(EVH1) and KRAS and suggest a mechanism by which this interaction is regulated during normal cell signaling and in cells with activated receptor tyrosine kinase oncogenes. The neurofibromin-SPRED1 interaction interface provides a rationale for mutations observed in Legius syndrome.

## Graphical Abstract

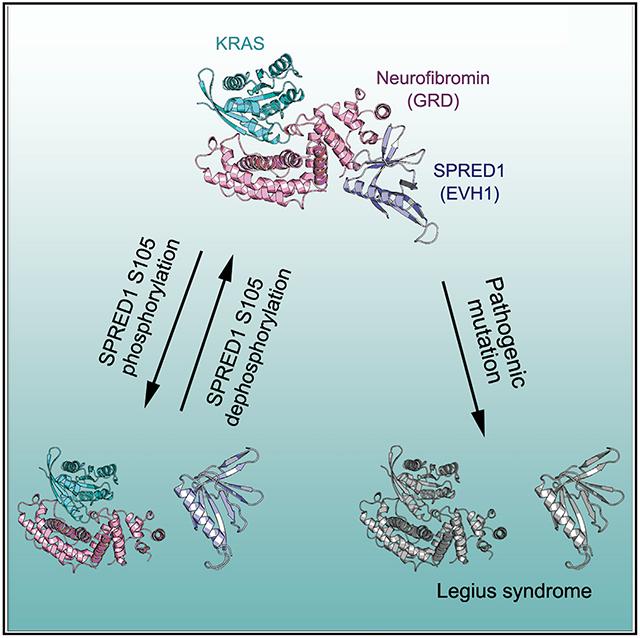

## INTRODUCTION

The *NF1* tumor suppressor gene encodes the cytoplasmic protein neurofibromin that acts as a RAS-specific GTPase-activating protein (GAP) and promotes the conversion of the active guanosine triphosphate (RAS-GTP) form to the inactive guanosine diphosphate (RAS-GDP) form, thereby downregulating its biological activity (Bos et al., 2007; Cherfils and Zeghouf, 2013; Scheffzek et al., 1997). Oncogenic mutations in *RAS* genes commonly lead to impaired GAP-mediated GTPase activity, which, in turn, results in constitutive activation of downstream signaling pathways (DeClue et al., 1992; Hobbs et al., 2016; Simanshu et al., 2017). Mutations in the *NF1* gene can also alter cellular growth and neural development, resulting in neurofibromatosis type 1, an autosomal dominant disorder that affects approximately one in 3,500 newborns worldwide (Philpott et al., 2017; Stumpf et al., 1988). Characteristic clinical features that are associated with neurofibromatosis type 1 include hyperpigmentary abnormalities of the skin (café-au-lait macules; CALM), inguinal/axillary freckling, skeletal abnormalities, Lisch nodules, and growth of benign peripheral nerve sheath tumors (neurofibromas) in the skin (Gerber et al., 2009). In addition to neurofibromatosis type 1, the *NF1* gene is often mutated in a variety of sporadic human malignancies, including glioblastoma, lung adenocarcinoma, acute myeloid leukemia, and ovarian and breast cancers (Blatt et al., 1986; Cancer Genome Atlas Research, 2008; Korf, 2000; Kresak and Walsh, 2016; Upadhyaya, 2011). The recently identified Legius syndrome, caused by germline mutations in the *SPRED1* gene, shares milder symptoms with neurofibromatosis type 1, including CALM, axillary freckling, and sometimes macrocephaly (Brems and Legius, 2013). Both syndromes are characterized as RASopathies, congenital developmental syndromes caused by germline mutations affecting the RAS/mitogen-activated protein kinase (MAPK) pathway (Aoki et al., 2016).

Neurofibromin (the protein is referred to here as NF1, encoded by the *NF1* gene) is a large, 320-kDa, multidomain protein that has been proposed to interact with multiple proteins (Donovan et al., 2002; Ratner and Miller, 2015). The GAP-related domain (GRD) located in the middle segment of NF1 is responsible for its RasGAP activity (Ballester et al., 1990; Martin et al., 1990; Xu et al., 1990). The GRD has a central portion called the minimal catalytic domain (GAPc) and an extra domain (GAPex) formed by residues that flank the GAPc. Although the apo structure of the NF1(GRD) was solved more than 2 decades ago (Scheffzek et al., 1998), our recent structural work on GMPPNP-bound KRAS in complex with the GAPc of NF1(GRD) provided structural insights into NF1-mediated GTPase activity (Rabara et al., 2019). In that study, we showed that, unlike G12 and Q61 mutants, G13 mutants of KRAS are frequently co-mutated with *NF1* and that NF1 hydrolyzes GTP directly in complex with KRAS-G13D. Besides RAS, SPRED (Sprouty-related, EVH1 domain-containing) proteins have been shown to bind NF1 directly and negatively regulate the RAS/MAPK pathway via this interaction (Stowe et al., 2012).

In mammals, the SPRED family consists of three members: SPRED1, SPRED2, and SPRED3 (Wakioka et al., 2001). All three variants can negatively regulate the RAS/MAPK pathway, and, unlike SPRED1 and SPRED2, SPRED3 has much weaker ERK-suppression activity (Kato et al., 2003). Gene-disruption studies have shown the functional similarity between SPRED1 and SPRED2, as SPRED1 and SPRED2 single-knockout mice were viable, while SPRED1/2 double-knockout mice were embryonically lethal (Taniguchi et al., 2007). The *SPRED1* gene encodes a 50-kDa SPRED1 protein, which comprises an N-terminal Ena/VASP Homology 1 (EVH1) domain and a C-terminal Sprouty-related domain (SPR) separated by a central c-Kit binding domain (KBD).

The EVH1 domain of SPRED1 interacts with NF1 (Stowe et al., 2012; Wakioka et al., 2001). This interaction is essential for NF1’s ability to suppress RAS activity. Mutations that cause Legius syndrome disrupt SPRED1-NF1 binding (Führer et al., 2019). The SPR domain undergoes palmitoylation, causing SPRED proteins to localize in the membrane fraction (Nonami et al., 2005). Epidermal growth factor (EGF) stimulation or galectin-1-mediated induction of RAF dimers has also been shown to translocate SPRED1 to the plasma membrane (Siljamäki and Abankwa, 2016). Pathogenic mutations in the SPR domain prevent membrane localization of the SPRED-NF1 complex (Stowe et al., 2012). Based on these data, a model was proposed in which SPRED proteins act as chaperones that are necessary to translocate NF1 to RAS on the plasma membrane (McClatchey and Cichowski, 2012; Stowe et al., 2012). This model explains why Legius syndrome resembles neurofibromatosis type 1 and explains the role of SPRED proteins in suppressing the RAS/MAPK pathway. Milder phenotypes associated with Legius syndrome relative to neurofibromatosis type 1 may be due to partial redundancy between SPRED1 and SPRED2 proteins. Recent studies have shown that SPRED1(EVH1) interacts with NF1(GRD) via the GAPex domain of NF1 and does not interfere with RAS binding or RasGAP activity (Dunzendorfer-Matt et al., 2016; Hirata et al., 2016). However, the structural details of the SPRED1-NF1 complex and the effects of the pathogenic mutations observed in Legius syndrome on SPRED1’s interaction with NF1 remain to be clarified.

The model in which SPRED proteins are essential for NF1’s ability to inactivate RAS raises questions of how these interactions are regulated and what is the structural basis for these interactions. In this study, we describe the structure of a ternary complex formed by the NF1(GRD) with the SPRED1 (EVH1 domain) and the active KRAS (GMPPNP bound). We show that SPRED1 and KRAS interact with NF1 via two separate interfaces, and we provide structural insights into how SPRED1 inhibits the RAS-ERK pathway by recruiting NF1 to RAS through the EVH1-GRD interaction. Our study also provides a structural explanation for the pathogenic mutations in *SPRED1* and *NF1* responsible for Legius syndrome and neurofibromatosis type 1, and it explains why SPRED1 interacts with NF1 GAP but not RASA1 GAP. Furthermore, we report that oncogenic EGF receptor (EGFR) disrupts the interaction between SPRED1 and NF1 by promoting phosphorylation on serine 105 within the EVH1 domain. These data suggest that oncogenic signals prevent NF1 from downregulating RAS, thus allowing sustained signaling without negative feedback.

## RESULTS

### Crystal Structure of NF1(GRD) in Complex with SPRED1(EVH1) and GMPPNP-Bound KRAS

**Figure 1.**
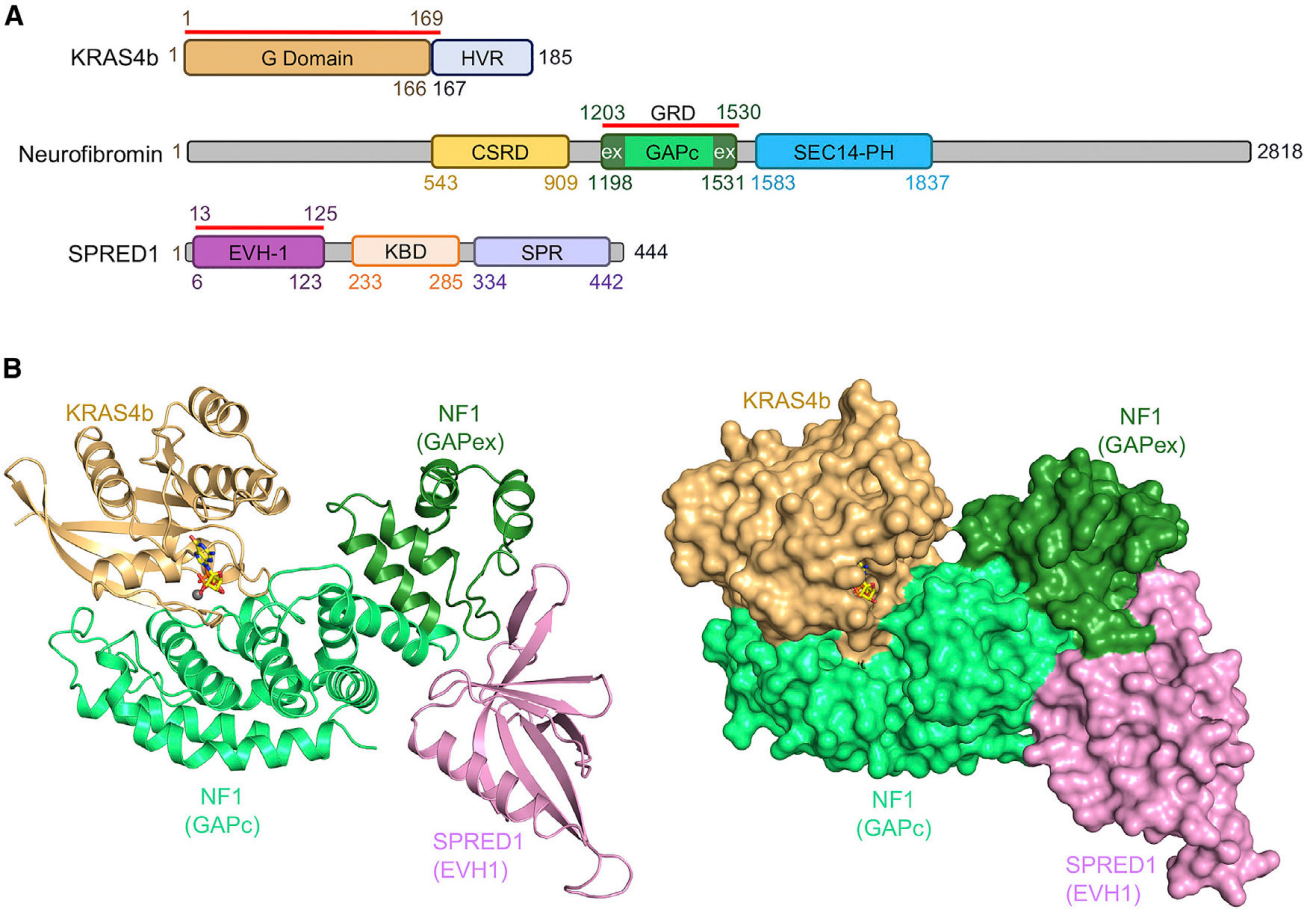
Domain Organization and Overall Structure of the Ternary Complex Formed by Neurofibromin (GRD), SPRED1(EVH1), and KRAS Proteins (A) Domain organization of KRAS, neurofibromin (NF1), and SPRED1 showing the presence of various domains in the full-length protein. The red line drawn above each protein indicates the domains involved in complex formation and used for our structural studies. In NF1, the GAPc and GAPex regions are highlighted in light and dark green, respectively. (B) The overall structure of the complex formed by GMPPNP-bound KRAS (light brown), NF1(GRD) (light green), and SPRED1(EVH1) (light pink) proteins. In the left panel, the ternary complex is indicated in ribbon representation, whereas in the right panel, proteins are indicated in surface representation. In both panels, GMPPNP and Mg^2+^ bound to KRAS are indicated as sticks and spheres, respectively. See also Figure S1 and Table S1.

To gain structural insight into how neurofibromin interacts with SPRED1 without affecting KRAS inactivation and to understand the structural basis of pathogenic *SPRED1* and *NF1* mutations in Legius syndrome and neurofibromatosis type 1, we attempted to solve the structure of the ternary complex composed of NF1(GRD), SPRED1(EVH1), and GMPPNP-bound KRAS (Figure 1A). Binding affinity measured using isothermal titration calorimetry (ITC) showed a dissociation constant (K_D_) of 224 nM between SPRED1(EVH1) and NF1(GRD), as well as a K_D_ of 1.3 μM between GMPPNP-bound KRAS and NF1(GRD) (Figures S1A and S1B). Since the Q61L mutant of KRAS binds to NF1(GRD) with 5-fold higher affinity than wild-type KRAS, we carried out crystallization of the ternary complex using wild-type as well as the Q61L mutant of KRAS (Figure S1C) and solved the crystal structures at resolutions of 2.75 Å and 2.55 Å, respectively (Table S1). The NF1, SPRED1, and KRAS domains used for the crystallization are highlighted in Figure 1A. The overall structure of the ternary complex is indicated in ribbon and surface representation in Figure 1B. As seen in the structure, NF1(GRD) acts as a scaffold and uses different interfaces to interact with KRAS and SPRED1. We see no interaction between KRAS and SPRED1, as suggested earlier (Dunzendorfer-Matt et al., 2016). NF1(GRD) shows an all-helical, crescent-shaped structure that interacts with KRAS primarily through its GAPc region, which forms the “belly” part of the crescent. In contrast, the residues that flank the GAPc region form the GAPex region present at one end of the crescent, which is the major interaction domain for SPRED1(EVH1) (Figure 1B), as proposed previously based on biochemical studies (Dunzendorfer-Matt et al., 2016; Hirata et al., 2016). Besides NF1(GAPex), the GAPc region of NF1(GRD) is also involved in the NF1-SPRED1 interaction. The structure of the ternary complex obtained with the KRAS-Q61L mutant aligns precisely with the ternary complex structure solved with wild-type KRAS (Figures S1D and S1E). Unlike the previously solved apo-NF1(GRD) structure where more than 20% of the residues were disordered (Scheffzek et al., 1998), most of the NF1(GRD) residues are observed in the structures described here, except residues C1465–S1476 as well as S1502–R1512, for which weak electron density was observed. The structural superposition of NF1(GRD) in the ternary complex and apo-NF1(GRD) (PDB: 1NF1) shows no structural rearrangement at the NF1-KRAS interface, suggesting that KRAS binding does not cause any major allosteric effects on NF1.

### Structure-Guided Mutational Analysis Confirms Key Residues Involved in Neurofibromin-KRAS Interaction

**Figure 2.**
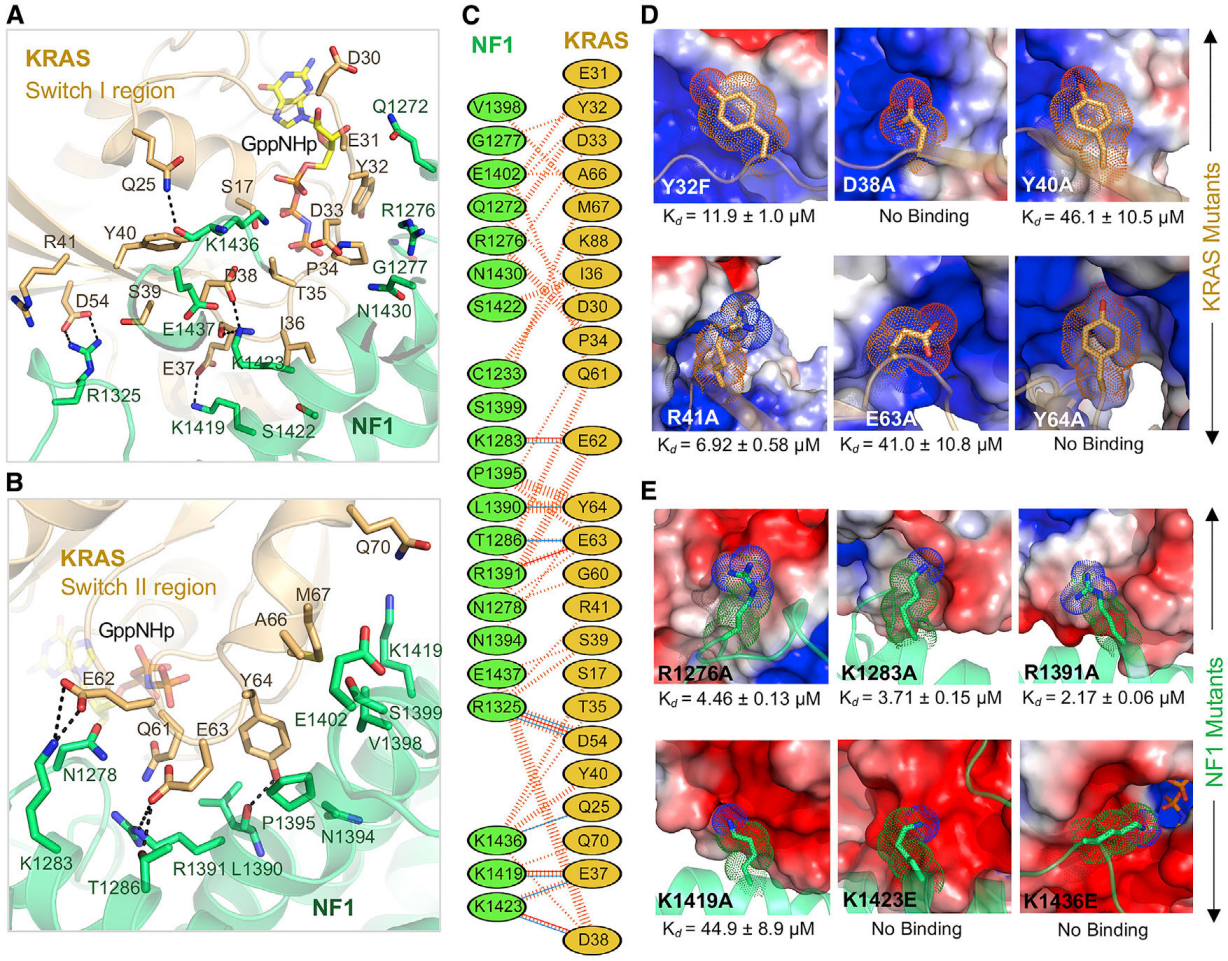
Structural Analyses of the NF1-KRAS Interaction Interface in the Ternary Complex and the Impact of Point Mutations in KRAS and NF1 on the NF1-KRAS Interaction (A and B) Enlarged view of the NF1-KRAS interaction interface formed by residues present in the (A) switch I and (B) switch II regions of KRAS. KRAS and NF1 are colored light brown and green, respectively. The nucleotide GMPPNP and residues that participate in the protein–protein interaction are indicated in stick (yellow) representation. Intermolecular hydrogen bonds and salt bridges are indicated by dashed black lines. (C) Schematic representation of the NF1-KRAS interaction interface as identified by PDBSum (http://www.ebi.ac.uk/thornton-srv/databases/cgi-bin/pdbsum/GetPage.pl?pdbcode=index.html). The interactions are indicated using the following notation: hydrogen bonds, blue solid lines; salt bridge, red solid lines; non-bonded contacts, striped lines (width of the striped line is proportional to the number of atomic contacts). (D) Binding affinities (measured using ITC) for KRAS point mutants (stick representation) located at the NF1-KRAS interaction interface. NF1 is indicated in an electrostatic surface representation. (E) Binding affinities (measured using ITC) for NF1 point mutants (stick representation) located at the NF1-KRAS interaction interface. KRAS is indicated in an electrostatic surface representation. See also Figure S2.

At the NF1-KRAS interface, KRAS residues in and around the switch I and II regions–specifically, residues from Q25 to R41, and from D54 to Q70–interact with multiple NF1 residues spread across the shallow groove of the GAPc region of GRD (Figures 2A-2C and Table S2). The interaction between NF1 and KRAS appears to be stabilized mainly by polar contacts, including five salt-bridge interactions involving the following pairs: K1283(NF1) and E62(RAS), R1391(NF1) and E63(RAS), K1419(NF1) and E37(RAS), K1423(NF1) and D38(RAS), and R1325(NF1) and D54(RAS) (Figures 2A-2C, S2A, and S2B). The NF1-KRAS interface also contains hydrogen bonds formed by the side-chain atoms between R1325(NF1) and S39(RAS) and between T1286(NF1) and E63(RAS). Point-mutation studies suggest that the residues located in the central part of the NF1-KRAS interface play a more significant role in the complex formation than the residues located on the edge of the interface (Figures 2D, S2C, and S2D). When KRAS residues located at the edge of the interface, like Y32 or R41, were mutated to alanine, the binding affinity (measured by ITC) decreased from 1.3 μM to 11.9 μM and 6.9 μM, respectively. On the other hand, when the KRAS residues present in the central part of the interface, such as D38, Y40, E63, and Y64, were mutated to alanine, the binding affinity decreased approximately 40-fold for Y40A and E63A mutations and to undetectable binding for D38A and Y64A mutations. These results are consistent with the previous observation where D38Aand E63H mutations in HRAS result in significant loss of RASA1-mediated GAP activity (Gideon et al., 1992).

We observed a similar trend when NF1 residues located at the NF1-KRAS interface were mutated to alanine (Figures 2E and S2E). NF1 residue substitutions, such as R1276A (the catalytic arginine finger), K1283A, and R1391A (of the FLR motif and expected to co-stabilize switch II during the transition state), located on the edge of the interface, had a marginal effect on NF1-KRAS interaction. Previously, mutation of R1276 wa shown to result in complete loss of GAP activity (Ahmadian et al., 1997b; Sermon et al., 1998), whereas mutation of R1391 to Ala results in a 400-fold reduction in GAP activity compared to the wild-type NF1. Unlike R1276, which acts as an arginine finger, R1391 plays a structural role by stabilizing the catalytic pocket and plays a secondary role in GAP activity. Unlike the substitution of residues located on the edge of the interface, point mutants of NF1 residues present in the center of the protein-protein interface had a significant effect on NF1-KRAS interaction. The NF1 mutation K1419A, which abolishes the salt-bridge interaction with E37 on KRAS, showed 30-fold weaker affinity than wild-type NF1(GRD). Similarly, when NF1 residue K1436, which points the terminal nitrogen atom toward the gamma-phosphate of KRAS-GMPPNP, was mutated to alanine, the binding affinity decreased by approximately 20-fold. Previously, NF1-K1423 was shown to be mutated to Glu and Gln in neurofibromas and solid tumors, and these mutations were shown to result in 200- to 400-fold lower GAP activity than the wild-type (Li et al., 1992). When the NF1 lysine residues (K1423 and K1436) involved in polar contacts at the interface were mutated to negatively charged glutamate, the interaction between NF1 and KRAS was completely abolished.

In addition to polar interactions, the NF1-KRAS interface also contains hydrophobic interactions formed by nonpolar residues. The partly hydrophobic nature of the interface provides an explanation for the higher affinity of the KRAS-Q61L oncogenic mutant with NF1(GRD), even though this mutant is unable to hydrolyze GTP efficiently. In the ternary complex formed by KRAS-Q61L, the side chain of L61 forms hydrophobic interactions with L1390 from NF1. Unlike KRAS-Q61L, a KRAS-Q61R mutation would disrupt this hydrophobic interaction and cause charge repulsion with arginine finger NF1-R1276. This explains why the KRAS-Q61R mutant binds to NF1 with a much lower affinity than KRAS-Q61L (Rabara et al., 2019).

### The Active Site Pocket in Neurofibromin-KRAS(GMPPNP) Complex and Comparison with RASA1-HRAS(GDP-AlF_3_) Complex

**Figure 3.**
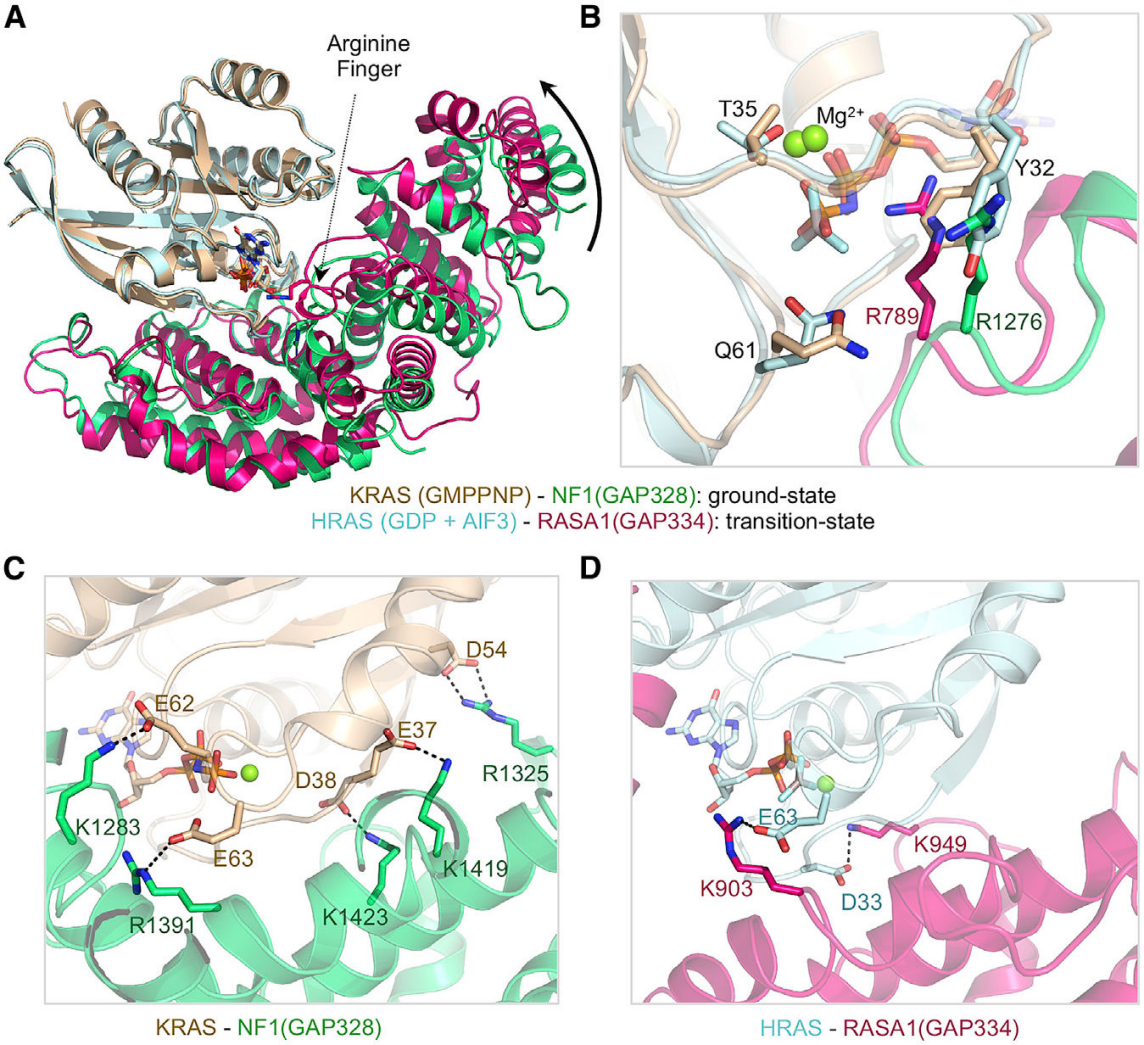
Structural Comparison between Ground-State and Transition-State Structures of RAS-RasGAP Complexes (A) The ground-state structure of GMPPNP-bound KRAS (light brown) in complex with NF1(GRD) (light green) superposed over the transition-state structure of GDP+AlF_3_-bound HRAS (cyan) in complex with RASA1(GRD) (hot pink). Structures were superposed using the RAS molecules present in these complexes. (B) Enlarged view of the active site pocket showing structural changes that occur between the ground state and the transition state. (C and D) Protein-protein interaction interface highlighting the presence of salt-bridge interactions in (C) NF1-KRAS and (D) RASA1-HRAS complexes. The color coding is the same as in (A).

Unlike the previously solved structure of the RASA1-HRAS complex in which a GDP-AlF_3_ module was used as a transition-state mimic (Scheffzek et al., 1997), the ternary complex described here was obtained in the presence of a slowly hydrolysable GTP analog, GMPPNP (GppNHp), corresponding to a ground-state conformation of the NF1-KRAS complex. This allows a comparison between the transition-state structure of HRAS-RASA1(GAP334) and the ground-state structure of KRAS-NF1(GAP328). Despite relatively low sequence identity between the GRD of RASA1 and NF1, the two structures share a common fold with a root-mean-square deviation (RMSD) of 1.7 Å (for the Cα atoms) and show a similar mode of binding to KRAS, suggesting that these two GAP proteins carry out GTPase activity in KRAS through a similar mechanism. In the ground-state structure, RAS-Y32 occupies the position of the arginine finger NF1-R1276 (R789 in RASA1) that points away from the active site (Figures 3A and 3B). Structural superposition of the ground- and transition-state complexes suggest that the GAPex region of NF1(GRD) undergoes a rotation of approximately 8.5° toward KRAS while shifting from the ground-state to a transition-state conformation (Figure 3A). Similar structural changes were observed in the ground-state structure of the NF1-GAPc domain in complex with wild-type and G13D mutant KRAS (Rabara et al., 2019). Structural transition from the ground state to the transition state would rearrange RAS-Y32 and arginine finger NF1-R1276 by swapping their positions. This enables the arginine finger to interact with the beta and gamma phosphates of GMPPNP and stabilize the negative charge in the active site pocket. Similar rotation and conformational changes, allowing effective placement of the arginine finger in the active site pocket, have been observed in Rho GTPases where structural information is available for both ground and transition states (Rittinger et al., 1997a, 1997b). The structural comparison between NF1-KRAS(GMPPNP) and RASA1-HRAS(GDP-AlF_3_) complexes showed the presence of five strong salt-bridge interactions in the NF1-KRAS complex spanning the protein-protein interface, whereas the RASA1-HRAS complex has only two such interactions at its interface (Figures 3C and 3D). It is tempting to speculate that the additional salt bridges present at the NF1-KRAS interface contribute to the higher affinity interaction of KRAS with NF1 compared to RASA1 GAP (Ahmadian et al., 1997a).

### The SPRED1-Neurofibromin Interface and a New Mode of Protein-Protein Interaction Formed by the EVH1 Domain

**Figure 4.**
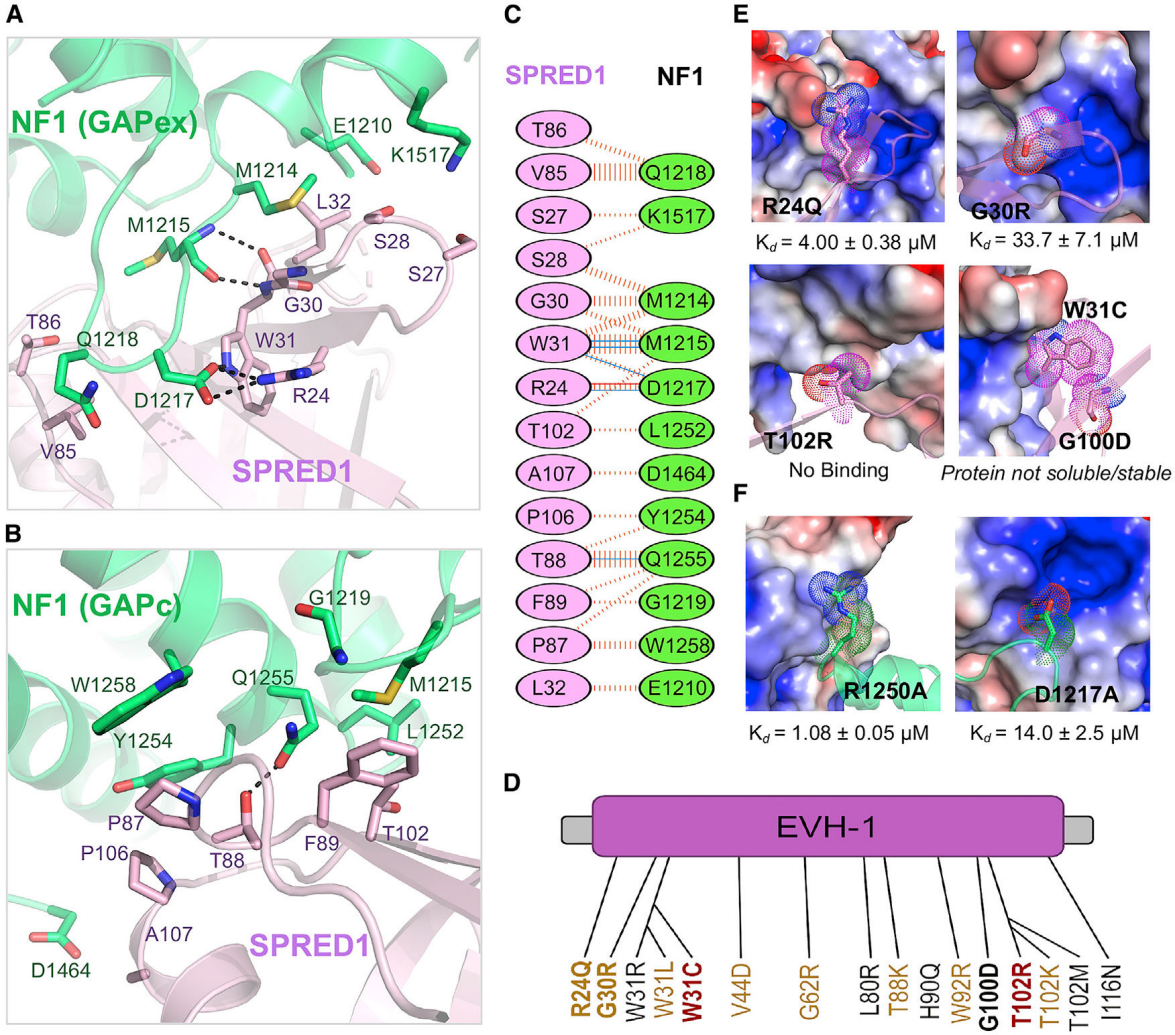
Details of the SPRED1-NF1 Interaction Interface in the Ternary Complex and the Impact of Pathogenic Mutations in SPRED1 and NF1 on the SPRED1-NF1 Interaction (A and B) Enlarged view of the SPRED1(EVH1)-NF1(GRD) interface formed by residues present in the (A) GAPex and (B) GAPc regions of NF1 in the ternary complex. SPRED1(EVH1) and NF1(GRD) are colored light magenta and green, respectively. The residues that participate in the protein-protein interaction are indicated in stick representation. Intermolecular hydrogen bonds and salt bridges are indicated by dashed black lines. (C) Schematic representation of the SPRED1-NF1 interaction interface as identified by PDBSum (http://www.ebi.ac.uk/thornton-srv/databases/cgi-bin/pdbsum/GetPage.pl?pdbcode=index.html/). The interactions are indicated in colors using the notation described for Figure 2C. (D) The missense mutations reported in the SPRED1(EVH1) domain in Legius syndrome. The text color represents the mutation type: red, confirmed pathogenic; yellow, suspected pathogenic; black, unclassified or uncertain. The mutations tested in this study are indicated in bold. (E) Binding affinities (measured using ITC) for Legius syndrome pathogenic mutations (stick representation) in SPRED1. NF1 is indicated in an electrostatic surface representation. (F) Binding affinities (measured using ITC) for neurofibromatosis type 1 pathogenic mutations (stick representation) in NF1. SPRED1 is shown in an electrostatic surface representation. See also Figure S3.

**Figure 5.**
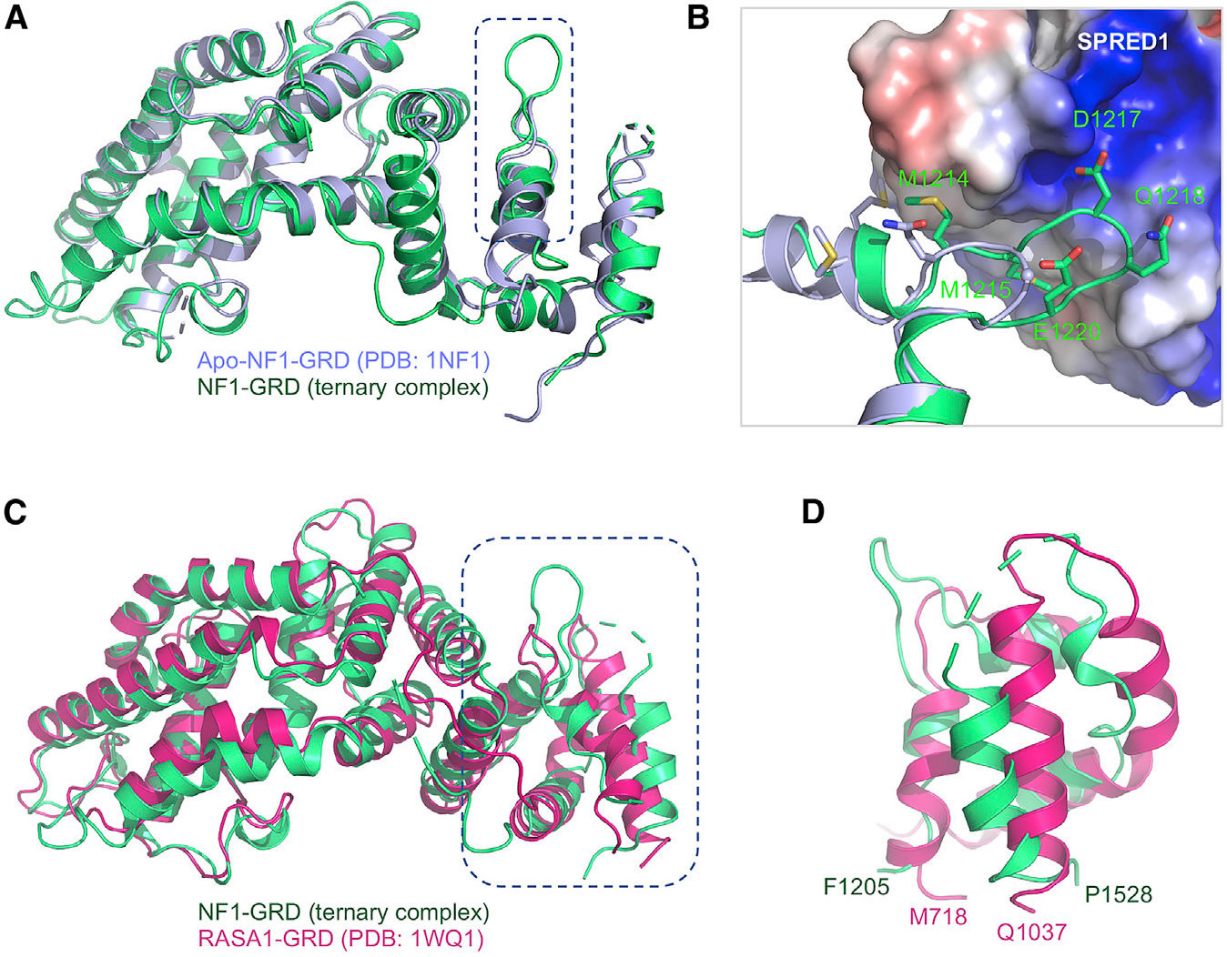
Structural Changes in NF1 upon Binding to SPRED1, along with Comparison of the GAPex Domains of NF1 and RASA1 (A) The NF1(GRD) (light green) present in the ternary complex superposed on the previously solved structure of the apo-form of NF1(GRD) (light blue). (B) Enlarged view of the GAPex region of NF1 that undergoes conformational change upon binding to SPRED1. SPRED1 is shown in an electrostatic surface representation, whereas NF1(GAPex) from the apo and ternary complexes are indicated in ribbon representation and colored light blue and green, respectively. The residues that undergo conformational changes are indicated in stick representation. (C) The NF1(GRD) (light green) present in the ternary complex superposed on the previously solved structure of RASA1(GRD) (hot pink). (D) Enlarged view of the superposed structures shown in (C), highlighting the structural differences between the GAPex region of NF1 and RASA1. See also Figure S4.

Previous biochemical studies suggested that the EVH1 domain of SPRED1 interacts with the GAPex domain of NF1 (Dunzendorfer-Matt et al., 2016; Hirata et al., 2016). In the ternary complex structure described here, the SPRED1(EVH1) domain interacts with both the GAPex and the GAPc regions of NF1(GRD). The overall structure of SPRED1 resembles the apo structure of SPRED1 solved previously from *Xenopus tropicalis* (RMSD = 1.04 Å for Cα atoms) and *Homo sapiens* (RMSD = 0.94 Å for Cα atoms). The total surface area buried upon SPRED1(EVH1)-NF1(GRD) complex formation is 849.8 Å^2^ for SPRED1(EVH1) and 887.3 Å^2^ for NF1(GRD), accounting for 12.5% (SPRED1) and 5.4% (NF1) of the total surface areas. The N-terminal residues L1211–E1220 of the NF1-GAPex domain undergo a structural change to form a long, finger-like loop that inserts itself into the pocket present on SPRED1 (Figures 4A, 4B, 5A, and 5B). Residue D1217 in NF1 forms a salt bridge with a conserved arginine (R24) in SPRED1, and the SPRED1 residue W31 forms hydrogen bonds with NF1 residues D1217 and M1215 (Figure 4C). The main-chain atoms of M1215 in NF1 form hydrogen bonds with the backbone atoms of G30 and W31 from SPRED1. Unlike the residues present at the N-terminal end of NF1(GRD), the residues located at the C terminus have less direct interaction with SPRED1 and act as a structure-supporting module to maintain the overall architecture between GAPc and the GAPex domains. A hydrophobic interaction exists between the aliphatic part (alkyl chain before the terminal amino group) of NF1-K1517 and the Cβ carbon atom of residues S27 and S28 in SPRED1, and a weak hydrogen bond is also present between the side-chain atoms of K1517(NF1) and S27(SPRED1). The SPRED1 region forming the SPRED1-NF1 interface in the crystal structure is consistent with the recently reported chemical shift changes observed in the ^15^N-labeled SPRED1(EVH1) mutant when it was titrated with the unlabeled NF1(GRD) in an NMR experiment (Führer et al., 2019).

As mentioned earlier, the structure shows that the NF1-GAPc region also interacts with SPRED1(EVH1) and contributes to complex stabilization mainly via hydrophobic interactions (Figure 4B and Table S3). This part of the NF1-SPRED1 interface is formed by interaction among residues R1250–W1258 and S1363–H1366 from the NF1-GAPc region and residues V85–H91 and T102–A107 from the SPRED1(EVH1) domain, which includes a hydrogen bond between Q1255(NF1) and T88(SPRED1) (Figure 4B; Table S3). The EVH1 domain has previously been shown to recognize proline-rich peptides in partner proteins using the grooves present on its surface (Peterson and Volkman, 2009). Since there is no proline-rich motif in NF1(GRD), and SPRED1(EVH1) interacts with NF1(GRD) via a narrow cleft, our results show a new binding mode for EVH1-domain-containing proteins.

### Structural Implications of Pathogenic Mutations Seen in Legius Syndrome and Neurofibromatosis Type 1

Although many of the Legius-syndrome-associated mutations found in the *SPRED1* gene result in premature stop codons, those that result in non-truncating missense mutations are often located within the EVH1 domain (Brems et al., 2012) (Figure 4D). Considering the phenotypic similarity between Legius syndrome and neurofibromatosis type 1, as well as the observation that NF1 is recruited to the plasma membrane by SPRED1, it has been hypothesized that mutations in SPRED1(EVH1) could cause Legius syndrome due to an inability to form the NF1-SPRED1 complex (Stowe et al., 2012). Recently, this hypothesis was supported by mutational studies in SPRED1 and NF1 using the yeast two-hybrid system (Hirata et al., 2016). To understand the structural basis of the pathogenicity of Legius syndrome mutations, we mapped various mutations observed in this RASopathy on the ternary complex structure described here (Figure S3A). Most of the missense mutations observed in the SPRED1(EVH1) domain are located on the NF1-SPRED1 interface (Figure S3C). We mutated several of these residues in SPRED1 that cause Legius syndrome in order to test the relationship between the disease phenotype and NF1 binding by measuring the K_D_ using ITC (Figures 4E, S3B, and S3D). The mutation R24Q (Sumner et al., 2011), which abolishes the salt bridge with residue D1217 of NF1, shows a 20-fold weaker binding (Figures 4A, 4E, and S3D). Two other mutants, G30R (Sumner et al., 2011) and T102R (Messiaen et al., 2009), which disrupt a hydrophobic interaction by introducing a relatively large side chain and charged amino acid arginine, result in an over 100-fold decrease in K_D_ and undetectable binding, respectively, between SPRED1 and NF1 (Figures 4A, 4B, 4E, and S4D). We also checked two other mutants at the interface: W31C (Denayer et al., 2011) and G100D (Brems et al., 2012), which have been reported to abolish the interaction between SPRED1 and NF1 *in vivo* (Hirata et al., 2016; Stowe et al., 2012). These two mutants failed to yield soluble and stable proteins, suggesting that they are likely to reduce the stability of the EVH1 domain of SPRED1 and may not undergo proper protein folding (Figures 4C and 4E).

We also mapped various pathogenic mutations observed in neurofibromatosis type 1 diseases, which include L1208W, L1211R, D1217G, and deletion of M1215 at the N-terminal end, and L1490P, Q1494R/E, and G1498E at the C-terminal end of the GAPex region in NF1 (Figures S3A and S3E). Among these–except NF1 residues M1215 and D1217, which are located at the NF1-SPRED1 interface–all other hydrophobic residues are located in the core of the GAPex domain (Figure S3A). The mutations of these hydrophobic residues to residues with bulky or charged side chains are likely to destabilize the folding of the GAPex region. We examined the D1217A(NF1) mutation using our ITC binding assay, and this mutant showed more than 50-fold weaker affinity to SPRED1(EVH1) (Figures 4F and S3F). Previously, it has been shown that the deletion mutant of M1215 fails to coimmunoprecipitate with SPRED1 and to localize to the membrane upon SPRED1 overexpression (Dunzendorfer-Matt et al., 2016).

We also noticed that the NF1 mutation R1250Q seen in cancer patients is located at the NF1-SPRED1 interface. In our binding assay, the R1250A(NF1) mutant showed a 5-fold reduced binding with SPRED1(EVH1), suggesting its involvement in NF1-SPRED1 interaction (Figures 4F, S3E, and S3F). To find out which NF1 residues present at the NF1-SPRED1 interface play an important role in NF1-SPRED1 complex formation, we carried out our point-mutational study of NF1 residues such as M1214, Q1255, H1366, and K1517, as they form a key interaction at the interface. Single point mutation of these NF1 residues to alanine/valine had no significant effect on the binding affinity between NF1 and SPRED1, suggesting that these NF1 residues individually do not contribute significantly to NF1-SPRED1 interaction (Figures S3E and S3F).

### Why Doesn’t SPRED1 Bind to the RASA1 GAPex Domain?

Like SPRED1(EVH1), RASA1(GRD) contains a GAPex domain. Previous studies have shown that SPRED1 binds to NF1(GRD) but not RASA1(GRD) (Hirata et al., 2016; Stowe et al., 2012). To understand the structural basis for the lack of interaction between SPRED1 and RASA1, we carried out sequence and structural comparisons of GRD of NF1 and RASA1, focusing on the GAPex region. Sequence alignment of GRD of NF1 and RASA1 shows 30% sequence identity in the GAPc domain (Figure S4A). However, these two proteins share almost no sequence similarity in the GAPex domain. Most of the NF1-GAPex residues that interact with SPRED1 are not conserved in RASA1. Importantly, the region from G1216–G1219 in NF1 (including the NF1 residue D1217 that forms a salt bridge with R24 in SPRED1), which forms the loop to bind to SPRED1, is missing in RASA1 (Figures 5A, 5B, and S4A). This makes it unlikely that RASA1 will undergo a similar conformational change to bind to SPRED1. Structural alignment of NF1(GRD) from the ternary complex structure described here and RASA1(GRD) from the previously solved RASA1-HRAS complex shows that though the GAPc regions align well between these two proteins, the GAPex regions show significant structural differences (Figures 5C and 5D). Thus, sequence and structural differences between the GRD of NF1 and RASA1 provide a rationale for SPRED1 binding to NF1 but not RASA1 (Ahmadian et al., 1996; Hirata et al., 2016; Stowe et al., 2012).

### SPRED1 Phosphorylation Disrupts SPRED1-Neurofibromin Interaction

**Figure 6.**
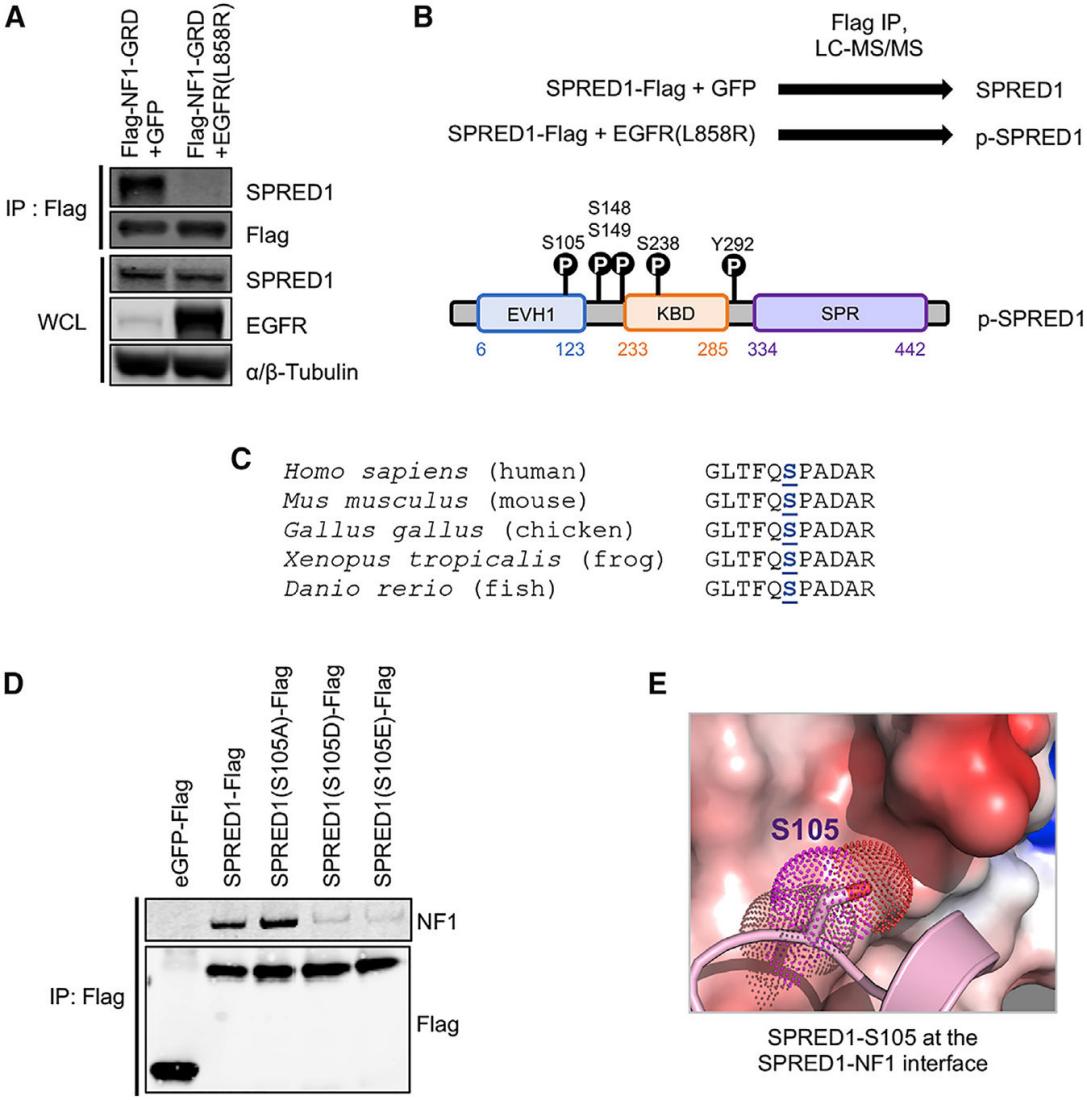
Identification of SPRED1 Phosphorylation that Disrupts SPRED1-NF1 Interaction (A) Proteins precipitated from extracts of HEK293T cells transiently transfected with FLAG-NF1 and EGFR(L858R) constructs were assessed by western blot for endogenous SPRED1 binding. WCL, whole-cell lysate; IP, immunoprecipitation. (B) Identification of SPRED1 phosphorylation sites downstream of EGFR(L858R) in HEK293T cells by SPRED1-FLAG and EGFR(L858R) transient transfection, anti-FLAG IP, and LC-MS/MS. (C) Amino acid sequence alignment of SPRED1 flanking serine 105 across indicated species shows evolutionary conservation. (D) Proteins precipitated from extracts of HEK293T cells transiently transfected with phosphomimetic and phosphodeficient SPRED1(S105) mutant constructs were assessed by western blot for endogenous NF1 binding. (E) Enlarged view of the NF1 electrostatic surface that interacts with SPRED1(S105). The S105E mutation results in a significant loss of binding affinity between SPRED1 and NF1. See also Figure S5.

Oncogenic receptor tyrosine kinases (RTKs) promote proliferation through sustained activation of the RAS/MAPK pathway. To do this, they presumably overcome negative feedback that normally turns this pathway off as part of the normal process of cellular signaling. NF1 has been implicated in this feedback process. We speculated that, in cells expressing constitutively active oncogenic tyrosine kinases, the interaction between NF1 and SPRED1 might be disrupted. Since gain-of-function EGFR mutations tend to be mutually exclusive with loss-of-function *SPRED1* and *NF1* mutations in lung adenocarcinoma (Collisson et al., 2014) (Figure S5A), we used oncogenic EGFR(L858R) as a model system. Expression of EGFR(L858R) disrupted SPRED1-NF1 binding, as shown by FLAG immunoprecipitation (IP) of the NF1(GRD) domain and western blot for endogenous SPRED1 in HEK293T cells (Figure 6A). To identify phosphorylation sites on SPRED1 that may disrupt SPRED1-NF1 binding, we co-expressed oncogenic EGFR(L858R) and SPRED1, immunoprecipitated SPRED1, and performed liquid chromatography-tandem mass spectrometry (LC-MS/MS) (Figure S5B). Five SPRED1 phosphorylation sites were identified (Figure 6B), including serine 105 in the EVH1 domain. Serine 105 is also in close proximity to threonine 102, which is mutated to arginine in Legius syndrome (Messiaen et al., 2009). SPRED1(T102R) is unable to bind NF1 and suppresses RAS-GTP following EGF stimulation (Stowe et al., 2012). Furthermore, in the crystal structure, both T102 and S105 are located in a hydrophobic region of SPRED1 that is part of the interacting area between NF1 and SPRED1; therefore, it is likely that the negative charge of the phosphate group on the hydrophobic surface of SPRED1 sterically repels NF1. The region flanking human SPRED1(S105) is also conserved across multiple species, from mouse to fish, further supporting the importance of this phosphorylation site (Figure 6C). To interrogate the potential disruption of NF1 binding by the phosphorylation of SPRED1(S105), we generated phosphomimetic and phosphodeficient SPRED1(S105) mutants and performed IP experiments. Phosphomimetic residues (aspartic and glutamic acid) decreased NF1 binding, while phosphodeficient (alanine) residues increased NF1 binding (Figure 6D). Using purified phosphomimetic SPRED1(S105D) and SPRED1(S105E) mutant proteins, we measured the binding affinity with NF1 (GRD) using ITC experiments. Mutation of SPRED1-S105 to Asp led to 10-fold weaker affinity, whereas mutation to Glu showed no measurable binding using ITC (Figure 6E). These experiments identify serine 105 as a critical phosphorylation site on SPRED1 that disrupts the SPRED1-NF1 complex.

### Phosphomimetic and Phosphodeficient SPRED1 Alters RAS-GTP Signaling after EGF Stimulation and K562 Proliferation

**Figure 7.**
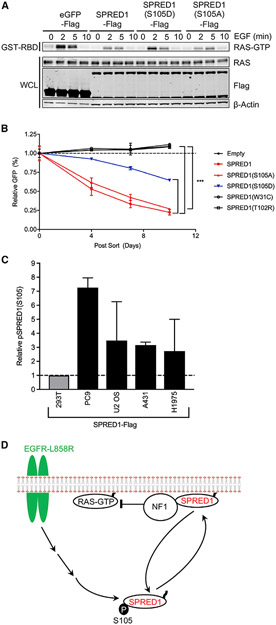
Phosphomimetic and Phosphodeficient SPRED1(S105) Alter RAS-GTP Signaling following EGF Stimulation and K562 Proliferation, along with a Model of SPRED1(S105) Phosphorylation (A) HEK293T cells were transiently transfected with indicated SPRED1-FLAG constructs, serum starved for 16 h, and stimulated with 10 ng/mL EGF for the indicted time points. Downstream signaling was then assessed by western blot and RAS-GTP pull-down assay. (B) K562 cells were infected with SPRED1-IRES-GFP, SPRED1-IRES-GFP mutants, and empty vector expressing retrovirus. Three days after infection, baseline GFP-positive cells were measured by flow cytometry and normalized to 1. During this competition assay, GFP-positive cells were monitored over time to measure the effect of SPRED1 expression on proliferation. The statistical significance of the difference between indicated samples was determined using a two-way ANOVA: ***p < 0.001. (C) Cancer cell lines were infected with SPRED1-FLAG-expressing retrovirus, selected with 1 μg/mL puromycin, anti-FLAG IP, and LC-MS/MS as described above. The y axis: MS1 chromatographic peak areas, arbitrary units relative to control. (D) Oncogenic EGFR-mediated SPRED1(S105) phosphorylation model. Phosphorylated SPRED1(S105) is unable to bind NF1 and inhibit RAS-GTP signaling. See also Figure S6.

In addition to the biochemical effect of SPRED1(S105) phosphorylation on NF1 binding, we were also interested in the biological effects of this process. We have previously demonstrated that SPRED1 overexpression decreased RAS-GTP following EGF stimulation in HEK293T cells and that SPRED1 mutants found in Legius syndrome were unable to decrease RAS-GTP (Stowe et al., 2012). As expected, the phosphomimetic SPRED1(S105D) is compromised in its ability to suppress RAS-GTP following EGF stimulation (Figure 7A). To determine whether the phosphomimetic SPRED1(S105D) also alters cancer cell proliferation, we used a cancer cell line dependent on RAS/MAPK signaling for proliferation with functional SPRED1-NF1 feedback. Recently, using an unbiased whole-genome CRISPRa screen, Boettcher et al. (2018) discovered that SPRED2 and NF1 overexpression inhibits K562 proliferation. K562 is a chronic myeloid leukemia (CML) cell line with the BCR-ABL oncogene, which is dependent on RAS-GTP for proliferation. Therefore, we expected that the K562 cell line would be an ideal model system to test the biological effects of phosphomimetic SPRED1(S105D), since in these cells, SPRED proteins, which are poorly phosphorylated on S105 (data not shown) can interact with NF1 to suppress growth. We infected K562 cells with SPRED1-IRES-GFP-expressing retrovirus and performed a competition assay between infected (GFP-positive) and uninfected (GFP-negative) cells (Figure 7B). Representative flow cytometry GFP histograms show similar infection rates and expression levels (Figure S6A). SPRED1 wild-type, GFP-positive cells were outcompeted by GFP-negative cells, while the empty vector controls were not. Likewise, the S105A mutant of SPRED1 behaved like wild-type, whereas the phosphomimetic SPRED1(S105D)-infected cells were compromised in their ability to inhibit proliferation. SPRED1 Legius syndrome patient mutants W31C and T102R that fail to bind NF1 did not affect proliferation (Figure 7B).

In addition to oncogenic EGFR(L858R)-mediated phosphorylation of SPRED1(S105) in HEK293T overexpression assays, we tested whether SPRED1 is phosphorylated in cancer cells with oncogenic EGFR mutations. We tested four EGFR mutant cancer cell lines and found that they all have elevated SPRED1(S105) phosphorylation (Figure 7C). While it is difficult to determine the stochiometry of phosphorylation using MS, we have estimated relative levels of phosphorylation by measuring the ratio of average measured retention times of modified and unmodified peptides (Figure S6B). We also analyzed phosphorylation on S107 and several other sites on SPRED1 in a wider panel of cancer cell lines expressing activated RTKs (Figures S6C and S6D). In addition to oncogenic EGFR, expression of other oncogenic RTKs, especially the D816V mutant of c-Kit, also increases SPRED1(S105) phosphorylation and other phosphorylation sites, broadening the scope of this finding.

## DISCUSSION

Although a number of interacting partners of neurofibromin have been reported in recent years (Ratner and Miller, 2015), the biological significance of these protein–protein interactions is still being investigated. RAS and SPRED are two families of proteins that bind to NF1, both at the GRD. While the ability of RAS proteins to interact with multiple effectors and regulators has been well documented (Simanshu et al., 2017), protein interactions with SPRED proteins are poorly understood. Here, we describe the structure of NF1(GRD) in complex with SPRED1(EVH1) and active KRAS and suggest a mechanism by which this interaction is regulated during normal cell signaling and in cells with activated RTK oncogenes. In the ternary complex structure of SPRED1-NF1-KRAS, GMPPNP-bound KRAS is bound to the GAPc domain of NF1(GRD) and provides the ground-state conformation of the NF1-KRAS complex. A comparison of the ground-state conformation of the NF1-KRAS complex with the transition-state conformation of the RASA1-HRAS complex shows conformational changes and rearrangement of the side chains in the active site pocket during the transition.

In humans, the *NF1* gene contains 60 exons and expresses multiple tissue-specific isoforms via alternative splicing with additional exons (9a, 10a-2, 23a, and 48a) (Barron and Lou, 2012). Among these NF1 isoforms, isoform types I and II differ by alternative splicing of exons 23a and 23b, respectively, present in the center of the GRD region. Unlike NF1 isoform II, which is described here, isoform I contains an additional 63-bp insertion (exon 23a) that encodes 21 amino acids in the center of the GRD region. Mapping the insertion site on the SPRED1-NF1-KRAS complex suggest that the inserted region does not directly affect either the NF1-SPRED1 interface or the NF1-KRAS interface. However, among the two protein-protein interfaces on NF1, the insertion site is proximal to the KRAS-binding site and thus likely to lead to steric hindrance, which may reduce its ability to inactivate RAS. NF1 isoform I is expressed in Schwann cells and is suggested to have a reduced GAP-stimulated GTPase activity (Hinman et al., 2014). This observation is supported by our *in* vitro measurements of protein affinities. The binding affinity of NF1 isoform I (GRD containing additional 23 residues) with SPRED1(EVH1) is similar to that of NF1 isoform II, whereas the affinity of NF1 isoform I for KRAS was 3-fold less than for NF1 isoform II (Figures S7A and S7B). Compared to NF1 isoform II, the relatively weaker interaction of NF1 isoform I with KRAS provides a rationale for the reduced RasGAP activity of NF1 isoform I. The specific expression patterns of the NF1 isoforms have been studied in several organs and cells and provide a basis for implicating differential expression of NF1 isoforms in the regulation of neuronal differentiation and development. Studies on sporadic colon, ovarian, and breast cancers have shown increased expression of isoform I relative to isoform II in tumor samples compared to normal tissue (Yap et al., 2014).

NF1, RASA1, and SynGAP are well characterized multidomain RasGAP proteins that contain a GRD domain with GAPex regions that flank the core catalytic region, GAPc. Although these RasGAP proteins share limited sequence and structural similarity in the GAPc region and use similar mechanisms for GAP-stimulated GTP hydrolysis, they use different domains and mechanisms for membrane anchoring. As shown previously, NF1-GAPex interacts with SPRED1 for membrane recruitment, whereas RASA1 and SynGAP use SH2 and PH domains, respectively, to anchor themselves to the membrane, presumably in response to specific signals (Kaplan et al., 1990; Pena et al., 2008; Stowe et al., 2012). For RASA1, these signals are thought to be phosphorylation of tyrosine kinases during growth-factor-induced signal transduction. After phosphorylation, RASA1 is recruited to the plasma membrane through its SH2 domains. It is then positioned to turn off RAS signaling through its GAP activity. It remains to be determined whether NF1 and SynGAP are regulated by analogous processes. The SPR domain of SPRED1 is essential for recruiting NF1 to the plasma membrane, but the signals that regulate this process are not yet understood. SPRED1 and SPRED2 bind to the c-Kit RTK (Wakioka et al., 2001): this may be critical to membrane recruitment. SPRED1 also binds to B-Raf and Galectin-1: these interactions regulate SPRED1 plasma membrane translocation and affect signaling from KRAS, but not HRAS (Siljamäki and Abankwa, 2016). Although the GRD domains of RASA1 and SynGAP contain GAPex regions, the differences in amino acid composition and tertiary structure provide a rationale for their inability to bind to the SPRED1(EVH1) domain. Further studies are needed to understand the role, if any, of the GAPex domain in RASA1 and SynGAP.

Previous biochemical studies have suggested that both the N- and C-terminal regions of NF1-GAPex are essential for SPRED1 binding (Dunzendorfer-Matt et al., 2016; Hirata et al., 2016). In the crystal structure, the N-terminal region of NF1-GA-Pex is the primary module that directly binds to SPRED1; the key residues, including M1214 and D1217, are all located in this region. The C-terminal region of GAPex interacts with SPRED1 indirectly, and the mutations in this region show a milder effect. Considering that NF1 fails to interact with SPRED1 when the C-terminal residues in the NF1-GAPex domain are deleted, the C-terminal residues likely serve to stabilize the GAPex domain.

SPRED proteins share significant sequence similarity in the EVH1 domain, and most of the EVH1 residues involved in SPRED1-NF1 interaction are conserved among them (Figures S4B and S4C). This explains why all three SPRED proteins are able to bind NF1, as shown previously (Stowe et al., 2012). The ability of three functionally overlapping SPRED proteins to bind NF1 is likely responsible for the relatively benign phenotype of Legius syndrome. Sequence alignment of SPRED family members shows that, unlike SPRED1 and SPRED2, SPRED3 has a smaller N terminus and contains a three-amino-acid insertion in the β4 strand. These insertions and deletions likely cause minor changes in the tertiary structure of SPRED3(EVH1) domain and, thus, may be responsible for the weaker ERK-suppression activity of SPRED3 compared with SPRED1 and SPRED2.

The EVH1 domain is a well-studied module that exists in multiple signaling proteins (Gertler et al., 1996; Reinhard et al., 1995). The EVH1 family of proteins has been classified into three major subfamilies: Ena/VASP, Homer/Vesl, and WASP (Ball et al., 2002; Beneken et al., 2000). The EVH1 domain recognizes proline-rich peptides, including FPPPP peptides (Ena/VASP), PPxxF (Homer/Vesl), and the LPPPEP motif (WASP) (Peterson and Volkman, 2009). A triad of aromatic residues, including a conserved tryptophan and phenylalanine as well as another hydrophobic residue, form a groove on the surface of the β strand, through which the proline-rich peptide can penetrate to bind EVH1. Because there is no proline-rich motif in NF1(GRD) and because SPRED1 contains a narrow cleft in contrast to other members of EVH1, it was proposed that SPRED1 binds to NF1 through a new interface (Harmer et al., 2005). The structural work presented here shows that the SPRED1(EVH1) domain interacts with NF1(GRD) in the ternary complex via a new mode of protein-protein interaction that may exist in other proteins containing an EVH1 domain. Interestingly, residue R24 of SPRED1 is conserved among all SPRED proteins but is replaced by tyrosine or isoleucine in the EVH1 domains of WASP and Homer/Vesl proteins, suggesting that this residue likely plays an important role in specificity toward different interacting partners of EVH1-containing proteins.

We also show that phosphorylation regulates the interaction between NF1 and SPRED1, which is essential for the RasGAP activity of NF1. Results presented here support our previous model predicting that the ability of NF1 to downregulate RAS requires interaction with SPRED proteins (Stowe et al., 2012). We now report that this interaction can be disrupted in cells in which the EGFR is activated and results in phosphorylation of S105 on SPRED1 (Figure 7D). SPRED1(S105) phosphorylation has been reported before in HeLa cells following synchronization and EGF stimulation as part of a phospho-proteome analysis (Sharma et al., 2014). In our studies, we also expressed an activated form of EGFR, EGFR(L858R), which occurs in several types of cancer, including non-small-cell lung adenocarcinoma. Furthermore, we detected phosphorylation of SPRED1 on S105 in several other cancer cell lines (PC9, U2OS, A431, and H1975). In cells expressing EGFR (L858R), phosphorylation on SPRED1-S105 disrupts high-affinity binding to NF1 and allows RAS proteins to remain in their active state without negative feedback. The Legius syndrome patient mutations SPRED1(W31C) and (T102R) are unable to inhibit cell proliferation, presumably because they fail to bind NF1. Likewise, the phosphomimetic mutant S105D is compromised in its ability to suppress growth. Our preliminary data suggest that CDK1 is the kinase responsible for this regulatory phosphorylation, consistent with the observation of Sharma et al. (2014) that inhibitory phosphorylation sites on CDK1 are downregulated following EGFR activation. Although the full significance of this discovery is beyond the scope of this study, it - raises the possibility that the direct interaction between SPRED proteins and NF1 is normally regulated in response to signals that determine levels of RAS-GTP and downstream signal transduction. This interaction may also be regulated by the levels of SPRED and NF1 proteins, additional posttranslational modifications, and association with other proteins, such as c-Kit, to which SPRED proteins bind. Considering the central importance of RAS signaling in cell biology, we believe that structural, biological, and biochemical analysis of Ras-GAPs with their regulators will continue to shed light on how RAS proteins are regulated in normal and pathologic conditions.

## STAR★METHODS

### RESOURCE AVAILABILITY

#### Lead Contact

Further information and requests for resources and reagents should be directed to and will be fulfilled by the Lead Contact, Dhirendra Simanshu (dhirendra.simanshu@fnlcr.nih.gov).

#### Materials Availability

Plasmids, proteins and other unique martials generated in this study will be made available on request by the Lead Contact with a completed Materials Transfer Agreement (MTA). A list of common commercial reagents used in this study can be found in the Key Resources Table.

#### Data and Code Availability

The X-ray structure data and atomic coordinates during this study have been deposited at the Protein Data Bank with accession codes PDB: 6V65 for KRAS(GMPPNP)-NF1(GRD)-SPRED1(EVH1) complex, and PDB: 6V6F for KRAS-Q61L(GMPPNP)-NF1(GRD)-SPRED1(EVH1) complex.

### EXPERIMENTAL MODEL AND SUBJECT DETAILS

The BL21 STAR (DE3) *E. coli* strain containing rare transfer RNAs (pRare plasmid, Cm^R^) was transformed with either KRAS or NF1 expression plasmids. Bacteria were cultured in Dynamite medium for protein expression (Taylor et al., 2017). SPRED1 was expressed in the baculovirus expression vector system using Tni-FNL cells (Talsania et al., 2019).

For cellular assay, SPRED1 was cloned into the pMIG (Addgene, #9044) MSCV-IRES-GFP vector. Retrovirus was generated using the VSV-G envelope expressing plasmid pMD2.G (Addgene #12259) and the packaging plasmid gag/pol (Addgene #14887). HEK293T cells were cultured in DMEM, high glucose (Thermo Fisher Scientific, 11965-084) and K562 were cultured in RPMI 1640 Medium (Thermo Fisher Scientific 118775-093). Media was supplemented with 10% FBS (Atlanta Biologicals S11550H) and Penicillin-Streptomycin (Thermo Fisher Scientific, 15140-122).

### METHOD DETAILS

#### *Escherichia coli* expression constructs

The DNA constructs for the expression of wild-type and point mutants NF1(GRD; 1198–1530 or 1203–1530) and KRAS4b(1–169) were created using the previously outlined protocols (Taylor et al., 2017) for expression in the format of His6-MBP-tev-POI (MBP, maltose-binding protein; tev, tobacco etch virus protease recognition sequence; POI, protein of interest). Two DNA constructs were used for the production of KRAS (1–169): one in the form of His6-MBP-tev-POI as described above, and one in the same form but under the control of the *tac* promoter rather than the T7 promoter. The DNA construct for the expression of NF1(1203–1530) was created using the previously outlined protocols (Taylor et al., 2017) for expression in the format of His6-MBP-tev-POI. *Insect expression constructs*. The DNA constructs for the expression of SPRED1(13–125) wild-type and point mutants for use in the baculovirus insect expression system were cloned in the baculovirus Destination vector pDest-635 (pFastBac1 with N-terminal His6 tag) in the form of His6-tev-POI using the previously described protocols (Sherekar et al., 2020).

#### Protein expression

All KRAS and NF1 proteins were expressed following protocols (Dynamite media protocol, 16°C induction) previously described for expression in *E. coli* (Taylor et al., 2017). Essentially, an overnight 37°C culture (non-inducing MDAG-135 medium) of the *E. coli* strain harboring the expression plasmid of interest, is used as seed culture to inoculate (2% v/v) expression-scale cultures of Dynamite medium. The expression culture is grown at 37°C until OD_600_ reaches 6-8, protein expression is induced with 0.5 mM IPTG, the culture is incubated at 16°C for 18-20 hours, and the cells are harvested by centrifugation. All SPRED1 proteins were expressed in the baculovirus expression vector system, as described previously (Agamasu et al., 2019). Essentially, the protein of interest is expressed from a viral promoter after infection of Tni-FNL cells with virus produced from engineered bacmids. Infected cells are incubated at 21 °C for 72 hr and harvested by centrifugation.

#### Protein purification

All proteins were purified in a similar manner. Specifically, frozen cell pellets were thawed and resuspended in lysis buffer containing 20 mM HEPES (pH 7.3), 300 mM NaCl, 1 mM TCEP, and 1:200 (v/v) protease inhibitor (PI) cocktail (P8849, Sigma-Aldrich, St. Louis, MO). All lysis and purification buffers for KRAS proteins were amended with 5 mM MgCl_2_. For *E. coli* expression material, cells were resuspended in 10 mL of lysis buffer per 1,000 optical density (OD) units. OD was measured at cell harvest at A_600_. For insect expression materials, cells were resuspended with 100 mL of lysis buffer/liter of expression culture. Homogenized cells were lysed by passing twice through an M-110EH Microfluidizer (Microfluidics Corp., Westwood, MA) at 9,000 psi for *E. coli* and 7,000 psi for insect cells. *E. coli* lysates were clarified by centrifugation at 7,900 x *g* (RCF average) for 90 minutes at 4°C. Insect cell lysates were clarified by centrifugation at 100,000 x *g* (RCF average) for 30 minutes at 4°C. Clarified lysates were filtered through 0.45 μM Whatman polyethersulfone syringe filters (GE Healthcare, Chicago, IL) and either used immediately or frozen at −80°C for future use. All proteins were purified on NGC chromatography systems (Bio-Rad Laboratories, Hercules, CA). Clarified lysates were thawed, adjusted to 35 mM imidazole, and loaded at 3 ml/minute onto immobilized metal affinity chromatography (IMAC) columns (Ni Sepharose High-Performance nickel-charged resin, GE Healthcare, Chicago, IL) equilibrated in an IMAC equilibration buffer (EB) of 20 mM HEPES (pH 7.3), 300 mM NaCl, 1 mM TCEP, 35 mM imidazole, and 1:1000 PI cocktail. The columns were washed to baseline with EB and proteins were eluted with a 20-column volume gradient from 35–500 mM imidazole in EB. Elution fractions were analyzed SDS-PAGE and Coomassie staining. Positive fractions were pooled, and His6-TEV protease (approximately 4 mg/ml lab stock prepared using plasmid #92414 from Addgene, and protocols referenced therein) was added at a 1:20 (v/v) protease:substrate ratio. The digestion proceeded while dialyzing to 20 mM HEPES (pH 7.3), 300 mM NaCl, and 1 mM TCEP for two hours at room temperature (approximately 22°C), then overnight at 4°C. The digested samples were processed by a second round of IMAC similar to the first round, except that the equilibration and wash buffers did not contain imidazole. Column flow-through and column wash were collected as fractions, and the columns were developed with a five-column volume gradient to 50 mM imidazole. After analysis of fractions by SDS-PAGE and Coomassie staining, appropriate fractions (the target proteins eluted either in the flow-through or at approximately 10–20 mM imidazole) were pooled and, if necessary, concentrated in 10K MWCO Amicon centrifugation units (MilliporeSigma, Burlington, MA). Pooled proteins were exchanged into final buffers (see below) by size-exclusion chromatography using appropriately sized columns packed with Superdex 75 resin (GE Healthcare, Chicago, IL). After SDS-PAGE and Coomassie staining were used to analyze fractions from the size-exclusion chromatography, appropriate fractions were pooled, concentrated in 10K MWCO Amicon centrifugation, filtered with a 0.22-μM syringe filter (low protein binding), assayed for protein concentration by measuring A_280_ (Nanodrop 2000C spectrophotometer, Thermo Scientific, Waltham, MA), dispensed as 0.25 mL aliquots, and snap-frozen in liquid nitrogen. The final buffer for all NF1 and SPRED1 proteins was 20 mM HEPES (pH 7.3), 150 mM NaCl, and 1 mM TCEP. The final buffer for KRAS proteins was 20 mM HEPES (pH 7.3), 150 mM NaCl, and 1 mM TCEP, which was amended with 1–5 mM MgCl_2_, depending on the protein concentration. The nucleotide exchange to replace GDP by GMPPNP in the purified wild-type and Q61L mutant of KRAS were carried out using the protocol described previously (Dharmaiah et al., 2019).

#### Complex formation, crystallization and data collection

To form a stable ternary complex of SPRED1-NF1-KRAS proteins, NF1(1198–1530), SPRED1(1–125), and KRAS(1-169) loaded with GMPPNP were mixed in a ratio of 1:1.2:1.2 and incubated on ice for an hour. This mixture was then loaded on the size-exclusion column to remove the unbound proteins, and peak fractions were pooled and concentrated for crystallization screening at a concentration of 12 mg/ml. Since both wild-type and Q61L mutant KRAS bind to NF1 with high affinity, we attempted the crystallization of the ternary complex using wild-type as well as Q61L mutant KRAS. Crystallization screenings were carried out using the sitting-drop vapor-diffusion method by mixing the ternary complex with an equal volume of reservoir solution. We obtained no crystallization hits at 20°C, so we next attempted crystallization at 4°C, which gave small crystals. Crystallization hits from initial screens at 4°C were optimized by systematically varying the pH, individual component concentrations, and the presence of additive and detergents. The best crystals of the ternary complex containing wild-type and Q61L mutant KRAS were obtained in 100 mM Tris (pH 7.8), 100 mM ammonium sulfate, 300 mM sodium formate, 3% PEG3350, and 3.5% PGA-LM in the presence of 10% detergent ANAPOE®-80 premixed with protein buffer. Sheet-like crystals were visible after seven days. Crystals were harvested for data collection and cryoprotected with a 25% (v/v) solution of ethylene glycol or glycerol mixed with crystallization solution before being flash-cooled in liquid nitrogen. Diffraction datasets were collected on the 24-ID-C/E beamlines at the Advanced Photon Source, Argonne National Laboratory. Crystallographic datasets were integrated and scaled using XDS (Kabsch, 2010). The crystal parameters and the data collection statistics are summarized in Table S1.

#### Structure determination and analysis

Structures of SPRED1-NF1-KRAS complexes were solved by molecular replacement using Phaser as implemented in the Phenix/CCP4 suite of programs, with a protein-only version of PDB entries 1NF1 (apo-structure of human NF1(GRD)), 3SYX (apo-structure of human SPRED1(EVH1)) as well as the structure of KRAS-GMPPNP (Adams et al., 2010; McCoy et al., 2007; Winn et al., 2011). The initial solution was refined using phenix.refine, and the resulting *Fo-Fc* map showed clear electron density for three proteins. The model was further improved using iterative cycles of manual model building in COOT (Emsley et al., 2010) and refinement using phenix.refine (Adams et al., 2010). After ligands were placed, potential sites of solvent molecules were identified by the automatic water-picking algorithm in COOT and phenix.refine. The positions of these automatically picked waters were checked manually during model building. Secondary structural elements were assigned using DSSP (https://swift.cmbi.umcn.nl/gv/dssp/). Figures were generated with PyMOL (Schrödinger, LLC), and surface electrostatics were calculated with APBS (Jurrus et al., 2018). Crystallographic and structural analysis software support was provided by the SBGrid Consortium (Morin et al., 2013).

#### Isothermal titration calorimetry (ITC) measurements

Binding affinities of point mutants of NF1, SPRED1, and KRAS were measured using ITC. Protein samples were prepared by dialyzing them in a buffer (filtered and degassed) containing 20 mM HEPES (pH 7.3), 150 mM NaCl, 5 mM MgCl_2_, and 1 mM TCEP. Before titration, all proteins were centrifuged at 14,000 x *g* for 5 minutes to remove any debris and air bubbles. Protein concentration was measured using absorbance at 280 nm. ITC experiments were performed in a MicroCal PEAQ-ITC (Malvern) out at 25°C using 19 injections of 2.2 μL administered at 150 s intervals. Data analysis was performed based on a binding model containing “one set of sites” by using a nonlinear least-squares algorithm incorporated in the MicroCal PEAQ-ITC analysis software (Malvern).

#### Plasmids and transient transfections

SPRED1 plasmids were generated as previously described (Stowe et al., 2012). Additional mutants were generated by PCR-directed mutagenesis and confirmed by sequencing. Transient transfection in HEK293T cells was performed with Lipofectamine 2000 Transfection Reagent (Thermo Fisher Scientific, 11668019) and Opti-MEM Reduced Serum Medium, with GlutaMAX Supplement Thermo Fisher Scientific, 51985091) following manufacturer’s recommendation. Fresh media was added 16 hours after transfection and cells were lysed the follow day in lysis buffer containing 20mM Tris (pH 7.5), 150 mM NaCl, 1 mM EDTA, 1% Triton X-100, 1 mM DTT, protease (Sigma Aldrich, P8340) and phosphatase inhibitors (Sigma Aldrich, P0044 and P5726). Immunoprecipitations were performed with 20 μl of EZview Red Anti-Flag M2 Affinity Gel clone M2 (Sigma-Aldrich, F2426).

#### RAS-GTP and Fractionation Assays

RAS-GTP assays were performed with the RAS Pull-down Activation Assay Biochem Kit (Cytoskeleton, BK008). Cells were serum starved for 16 hours and stimulated with 10 ng/ul EGF (Thermo Fisher Scientific, PHG0311). Fractionation was performed using NE-PER Nuclear and Cytoplasmic Extraction Reagents (Thermo Fisher Scientific, 78835)

#### Antibodies

Flag 1:1,000 (Sigma, V8137), SPRED1 1:1,000 (Cell Signaling, 94063), α/β-Tubulin (Cell Signaling 2148), EGFR 1:200 (Santa Cruz Biotech, 1005, SC-03), Neurofibromin (Santa Cruz Biotech, sc-67), RAS 1:200 (Cytoskeleton, AESA02), β-Actin 1:10,000 (Sigma-Aldrich, A5441).

#### K562 Competition

SPRED1 was cloned into the pMIG (Addgene, #9044) MSCV-IRES-GFP vector. Retrovirus was generated using the VSV-G envelope expressing plasmid pMD2.G (Addgene #12259) and the packaging plasmid gag/pol (Addgene #14887). HEK293T cells were transfected with Lipofectamine 3000 Transfection Reagent (Thermo Fisher Scientific, L3000015) as described above. 16 hours after transfection fresh media was added containing ViralBoost Reagent (Alstem, #VB1000). 24 hours later virus was filtered (0.45 μM), polybrene (Sigma Aldrich, H9268) at 4 μg/ml. was added, and K562 cells were infected by spinfection at 2,000 RPM for 1 hour. GFP positive cells were analyzed on the Sony Cell Sorter SH900Z.

#### SPRED1 Mass Spectrometry

One 10 cm plate of HEK293T cells per condition was transfected with plasmids as above. Immunoprecipitations were carried out using 60 μl of anti-FLAG antibodies coupled to magnetic beads (Anti-FLAG M2 Magnetic Beads, Sigma, M8823). Rotating at 4°C for 2 hours and washed three times with TME. Two final washes with ice-cold 20 mM TrisHCl pH8 + 2 mM CaCl2 were carried out on ice. The beads were then resuspended in 9 μL of 20 mM Tris-HCl pH 8.0. The proteins were reduced by adding 0.4 μL of 100 mM DTT and incubating at room temperature for 30 min with agitation and alkylated by adding 0.6 μL of 100 mM iodoacetamide and incubating at room temperature for 10 min with agitation. Digestion with 500 ng of trypsin (Sigma Trypsin Singles, T7575) was carried out at 37°C overnight with mild agitation. The digest was stopped by adding formic acid to a final concentration of 2%. The samples were desalted using ZipTipu-C18 pipette tips (Millipore) according to the manufacturer’s protocol and reconstituted in 12 μL of 0.1% formic acid.

Five μL of each digest was analyzed by liquid chromatography tandem mass spectrometry (LC-MS/MS) on a Q Exactive Plus instrument (Thermo Fisher Scientific) online with Waters NanoAcquity UPLC system (Waters). Reversed-phase chromatography was performed on a 15 cm silica-C18 EasySpray column (Thermo Fisher Scientific) at 45°C with a binary buffer system (Buffer A = 0.1% formic acid in water; Buffer B = 0.1% formic acid in acetonitrile) and a flow rate of 400 nL/min. The sample was loaded at 2% B for 20 min followed by a 2%–60% B gradient over 60 min, followed by a brief wash at 80% B and equilibration at 2% B. The Q Exactive Plus instrument was operated in Full-MS/ddMS2 mode with one survey scan (350-1500 m/z, R = 70,000 at 200 m/z, AGC target of 3e6), followed by up to 10 data-dependent HCD MS2 scans (AGC target of 5e4, max IT 120 ms, R = 17,500 at 200 m/z, isolation window 4.0 m/z, NCE 25%, 4% underfill ratio, and 10 s dynamic exclusion). Raw data files were converted to peak list files using Proteome Discoverer v. 1.4 (Thermo Fisher Scientific) and searched using Protein Prospector (Baker and Chalkley, 2014; Chalkley et al., 2005) version 5.14.0 against human SwissProt database (Bairoch and Apweiler, 2000) downloaded on 07/29/2013 and corresponding random concatenated decoy database with default “ESI-Q-high-res” parameters, including up to two allowed missed cleavage sites, Carbamidomethyl-C constant modification, default variable modifications plus phosphorylation at STY, up to 3 modifications per peptide, and 20 ppm precursor mass and fragment mass accuracy. False discovery rate of < 1% was used as the cutoff for peptide expectation values. Quantitation of relative phosphorylation at the S105 site in SPRED1/2 was carried out in Skyline v 3.0 (MacLean et al., 2010) by quantifying MS1 precursor peak areas of the S105-containing peptides and normalizing them by the sum of abundances of all unmodified peptides detected in the same protein.

### QUANTIFICATION AND STATISTICAL ANALYSIS

Statistical significance was determined for differences in K562 proliferation comparisons between control, SPRED1 wild-type, and SPRED1 mutants using a two-way ANOVA using GraphPad Prism. ***p < 0.001. ITC Data analysis was performed based on a binding model containing “one set of sites” by using a nonlinear least-squares algorithm incorporated in the MicroCal PEAQ-ITC analysis software (Malvern) to give the binding constants (K_D_), reaction stoichiometry (N), enthalpy (ΔH) and entropy (ΔS) for the KRAS-NF1 interaction as well as NF1-SPRED1 interaction.

## Supplementary Material

1

## Figures and Tables

**Table:** KEY RESOURCES TABLE

REAGENT or RESOURCE	SOURCE	IDENTIFIER
Antibodies		
Flag 1:1,000	Sigma	V8137
SPRED1 1:1,000	Cell Signaling	94063
α/β-Tubulin 1:2,000	Cell Signaling	2148
EGFR 1:200	Santa Cruz Biotech	1005, SC-03
Neurofibromin 1:200	Santa Cruz Biotech	SC-67
RAS 1:200	Cytoskeleton	AESA02
β-Actin 1:10,000	Sigma-Aldrich	A5441
Bacterial and Virus Strains		
BL21 STAR (DE3)	Thermo Fisher	C601003
Tni-FNL insect cells	FNLCR	Tni-FNL
Baculovirus, Bac-to-Bac	Thermo Fisher	AcMNPV
Retrovirus: pMD2.G	Addgene	12259
Retrovirus: gag/pol	Addgene	14887
Biological Samples		
HEK293T	UCSF Cell and Genome Engineering Core	CCLZR076
K562	UCSF Cell and Genome Engineering Core	CCLZR466
Chemicals, Peptides, and Recombinant Proteins		
protease inhibitor (PI) cocktail	Sigma-Aldrich	P8849, P8340
ANAPOE®-80	Molecular Dimensions	APT080 500 ML
PGA-LM	Molecular Dimensions	MD2-250-108
PEG3350 (50% w/v)	Hampton Research	HR2-527
DMEM, high glucose	Thermo Fisher	11965-084
RPMI 1640	Thermo Fisher	118775-093
FBS	Atlanta Biologicals	S11550H
Penicillin-Streptomycin	Thermo Fisher	15140-122
Opti-MEM Reduced Serum Medium	Thermo Fisher	51985091
phosphatase inhibitors	Sigma Aldrich	P0044, P5726
EGF	Thermo Fisher	PHG0311
Nuclear and Cytoplasmic Extraction Reagents	Thermo Fisher	78835
Lipofectamine 3000 Transfection Reagent	Thermo Fisher	L3000015
ViralBoost Reagent	Alstem	VB1000
trypsin	Sigma Trypsin Singles	T7575
Critical Commercial Assays		
RAS Pull-down Activation Assay Biochem Kit	Cytoskeleton	BK008
Lipofectamine 2000 Transfection Reagent	Thermo Fisher	11668019
Deposited Data		
Crystal structure of KRAS-NF1-SPRED1	This study	PDB:6V65
Crystal structure of KRAS(Q61L)-NF1-SPRED1	This study	PDB:6V6F
Experimental Models: Cell Lines		
HEK293T	UCSF Cell and Genome Engineering Core	CCLZR076
K562	UCSF Cell and Genome Engineering Core	CCLZR466
Spred1(S105A)-Forward PrimerGGTCTTACGTTTCAAGCTCCTGCTGATGCTAGG	Sigma-Aldrich	N/A
Spred1(S105A)-Reverse PrimerCCTAGCATCAGCAGGAGCTTGAAACGTAAGACC	Sigma-Aldrich	N/A
Spred1(S105D)-Forward PrimerGTTTGGTCTTACGTTTCAACATCCTGCTGATGCTAGGGC	Sigma-Aldrich	N/A
Spred1(S105D)-Reverse PrimerGCCCTAGCATCAGCAGGATGTTGAAACGTAAGACCAAAC	Sigma-Aldrich	N/A
Spred1(S105E)-Forward PrimerGGTCTTACGTTTCAAGAACCTGCTGATGCTAG	Sigma-Aldrich	N/A
Spred1(S105E)-Reverse PrimerCTAGCATCAGCAGGTTCTTGAAACGTAAGACC	Sigma-Aldrich	N/A
Spred1(W31C)-Forward PrimerCTCAAGTGGTGGATGCTTACCACTTGGAGGG	Sigma-Aldrich	N/A
Spred1(W31C)-Reverse PrimerCCCTCCAAGTGGTAAGCATCCACCACTTGAG	Sigma-Aldrich	N/A
Spred1(T102R)-Forward PrimerCAAGAAGTTTGGTCTTAGGTTTCAAAGTCCTGCTG	Sigma-Aldrich	N/A
Spred1(T102R)-Reverse PrimerCAGCAGGACTTTGAAACCTAAGACCAAACTTCTTG	Sigma-Aldrich	N/A
Recombinant DNA		
pDZ2087 (Expresses TEV protease in *E. coli*)	Addgene	92414
pMIG	Addgene	9044
pMD2.G	Addgene	12259
SPRED1 expression plasmid for cellular assay	(Stowe et al., 2012)	N/A
Software and Algorithms		
XDS	(Kabsch, 2010)	N/A
Phaser	(McCoy et al., 2007)	N/A
Phenix	(Adams et al., 2010)	N/A
COOT	(Emsley et al., 2010)	N/A
DSSP	https://swift.cmbi.umcn.nl/gv/dssp/).	N/A
PyMOL	Schrödinger, LLC	Ver: 2.3.2
SBGrid Consortium	(Morin et al., 2013)	N/A
PDBSum	http://www.ebi.ac.uk/thornton-srv/databases/cgi-bin/pdbsum/GetPage.pl?pdbcode=index.html/	N/A
MicroCal PEAQ-ITC analysis software	Malvern	N/A
Proteome Discoverer	Thermo Fisher	Ver: 1.4
Protein Prospector	(Baker and Chalkley, 2014; Chalkley et al., 2005)	Ver: 5.14.0
SwissProt database	(Bairoch and Apweiler, 2000)	N/A
Skyline v 3.0	(MacLean et al., 2010)	V 3.0
Other		
Immobilized metal affinity chromatography columns	GE Healthcare	18-1174-40
NGC chromatography system	Bio-Rad Laboratories	NGC-100
10K MWCO Amicon centrifugation units	MilliporeSigma	UFC901008
MicroCal PEAQ-ITC	Malvern	N/A
Anti-FLAG antibodies coupled to magnetic beads	Sigma	M8823
Liquid chromatography tandem mass spectrometry	Thermo Fisher	N/A
Silica-C18 EasySpray column	Thermo Fisher	N/A

## Highlights

- Crystal structure of the SPRED1-NF1-KRAS complex
- The NF1(GRD) uses different interfaces to interact with KRAS and SPRED1
- The SPRED1-NF1 interface provides a rationale for mutations seen in Legius syndrome
- SPRED1 phosphorylation at S105 disrupts SPRED1-neurofibromin interaction
