# Supplementary material for: Structural Insights into the SPRED1-Neurofibromin-KRAS Complex and Disruption of SPRED1-Neurofibromin Interaction by Oncogenic EGFR
Source: Cell Rep. Author manuscript; Available in PMC 2020 Aug 19. (PMC7437355; doi:10.1016/j.celrep.2020.107909)
Supplement: 1 [file NIHMS1613940-supplement-1.pdf]

Cell Reports, Volume *32*

Supplemental Information

Structural Insights into the SPRED1-Neurofibromin-KRAS Complex and Disruption of SPRED1-Neurofibromin Interaction by Oncogenic EGFR

Wupeng Yan, Evan Markegard, Srisathiyanarayanan Dharmaiah, Anatoly Urisman, Matthew Drew, Dominic Esposito, Klaus Scheffzek, Dwight V. Nissley, Frank McCormick, and Dhirendra K. Simanshu

Structural insights into the SPRED1-neurofibromin-KRAS complex and disruption of SPRED1-neurofibromin interaction by oncogenic EGFR

Wupeng Yan[a,1], Evan Markegard[b,1], Srisathiyanarayanan Dharmaiah[a], Anatoly Urisman[b], Matthew Drew[a], Dominic Esposito[a], Klaus Scheffzek[c], Dwight V. Nissley[a], Frank McCormick[a,b,#], Dhirendra K. Simanshu[a,#]

SUPPLEMENTARY INFORMATION

- **Figure S1-S7**
- **Table S1-S3**

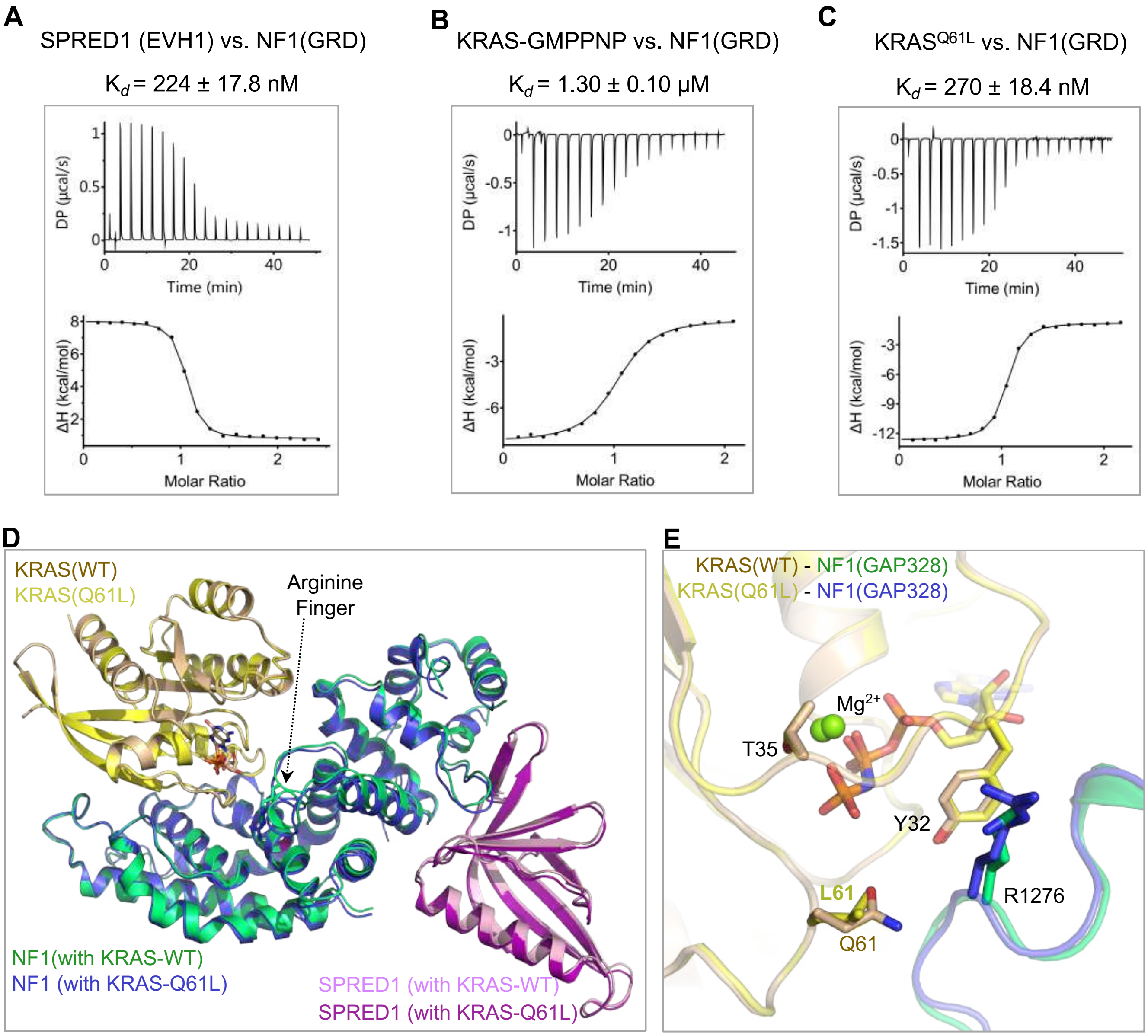

Figure S1. Intermolecular protein-protein interactions in KRAS-NF1(GRD)-Spred1(EVH1) complex and structural comparison between ternary complexes containing wild-type and Q61L-mutant of KRAS. (**A-C**) ITC titration experiment to measure the dissociation constant between (A) Spred1(EVH1) and NF1(GRD), (B) KRAS(WT)-GMPPNP and NF1(GRD), and (C) KRAS(Q61L)-GMPPNP and NF1(GRD). (**D**) Structural superposition of the ternary complexes containing wild-type and Q61L-mutant of KRAS. The proteins are shown in ribbon representation, whereas nucleotide GMPPNP and the arginine finger are shown in stick representation. (**E**) Enlarged view of the active site pocket in the ternary complexes containing wild-type and Q61L-mutant of KRAS. The arginine finger from NF1, nucleotide GMPPNP, and KRAS residues are shown in stick representation whereas Mg^{2+} are shown as spheres.

Related to Figure 1.

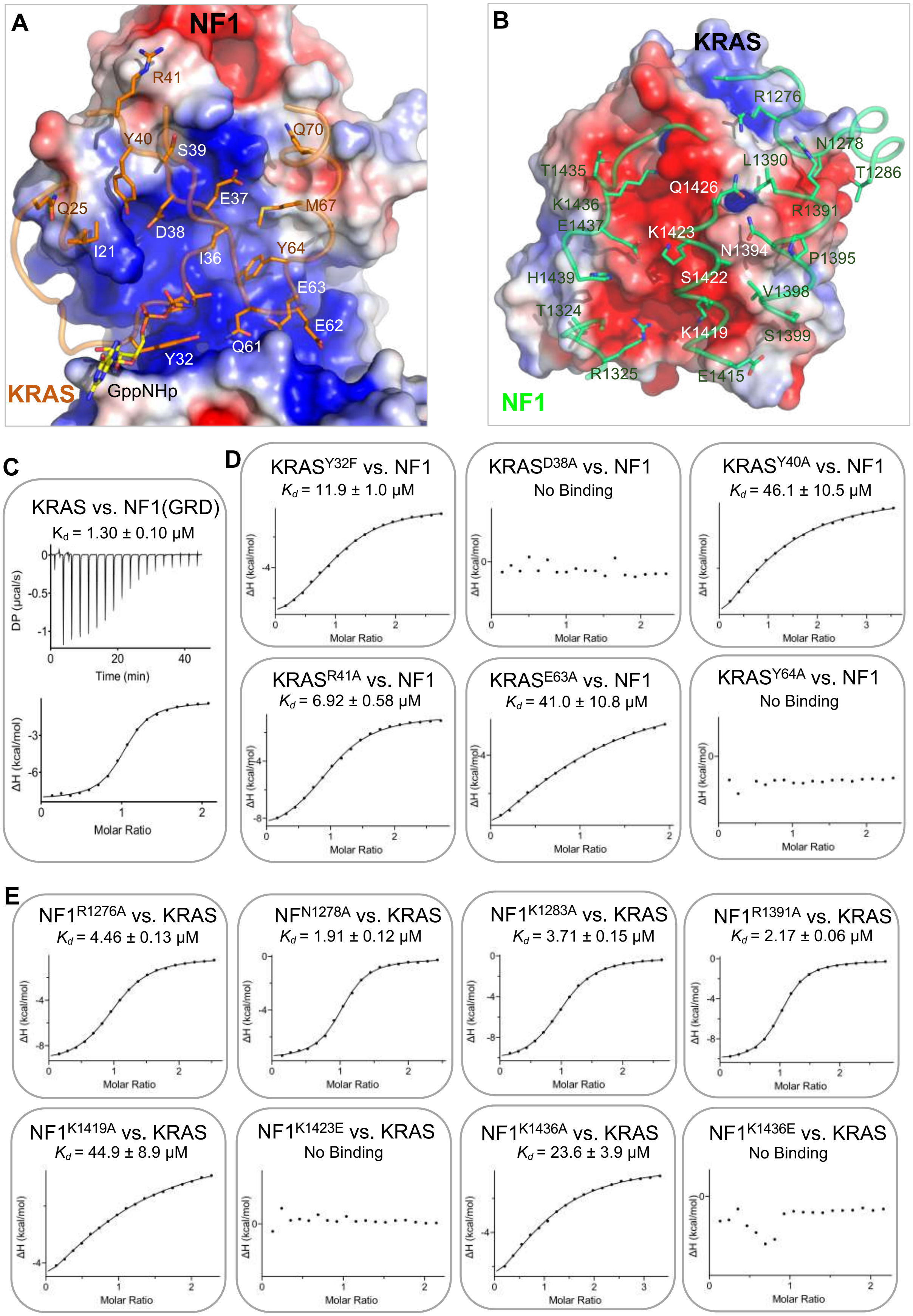

Figure S2. The KRAS-NF1(GRD) interaction interface and the impact of KRAS and NF1 mutations for residues involved in KRAS-NF1(GRD) interaction. (**A**) KRAS(GMPPNP)-NF1(GRD) interaction interface, with NF1 shown in electrostatic surface representation and the KRAS regions that participate at the interface shown in cartoon mode and colored brown. The KRAS

residues which are important for the interaction are highlighted in stick mode. **(B)** KRAS(GMPPNP)-NF1(GRD) interaction interface, with KRAS shown in electrostatic surface representation and the NF1(GRD) regions that participate at the interface shown in cartoon mode and colored green. The NF1 residues that are important for the interaction are highlighted in stick representation. **(C)** ITC titration experiment to measure the dissociation constant between KRAS(WT)-GMPPNP and NF1(GRD). **(D)** The dissociation constant of KRAS mutants with NF1(GRD) measured using ITC titration experiments. **(E)** The dissociation constant of NF1 mutants with KRAS(GMPPNP) measured using ITC titration experiments.

Related to Figure 2.

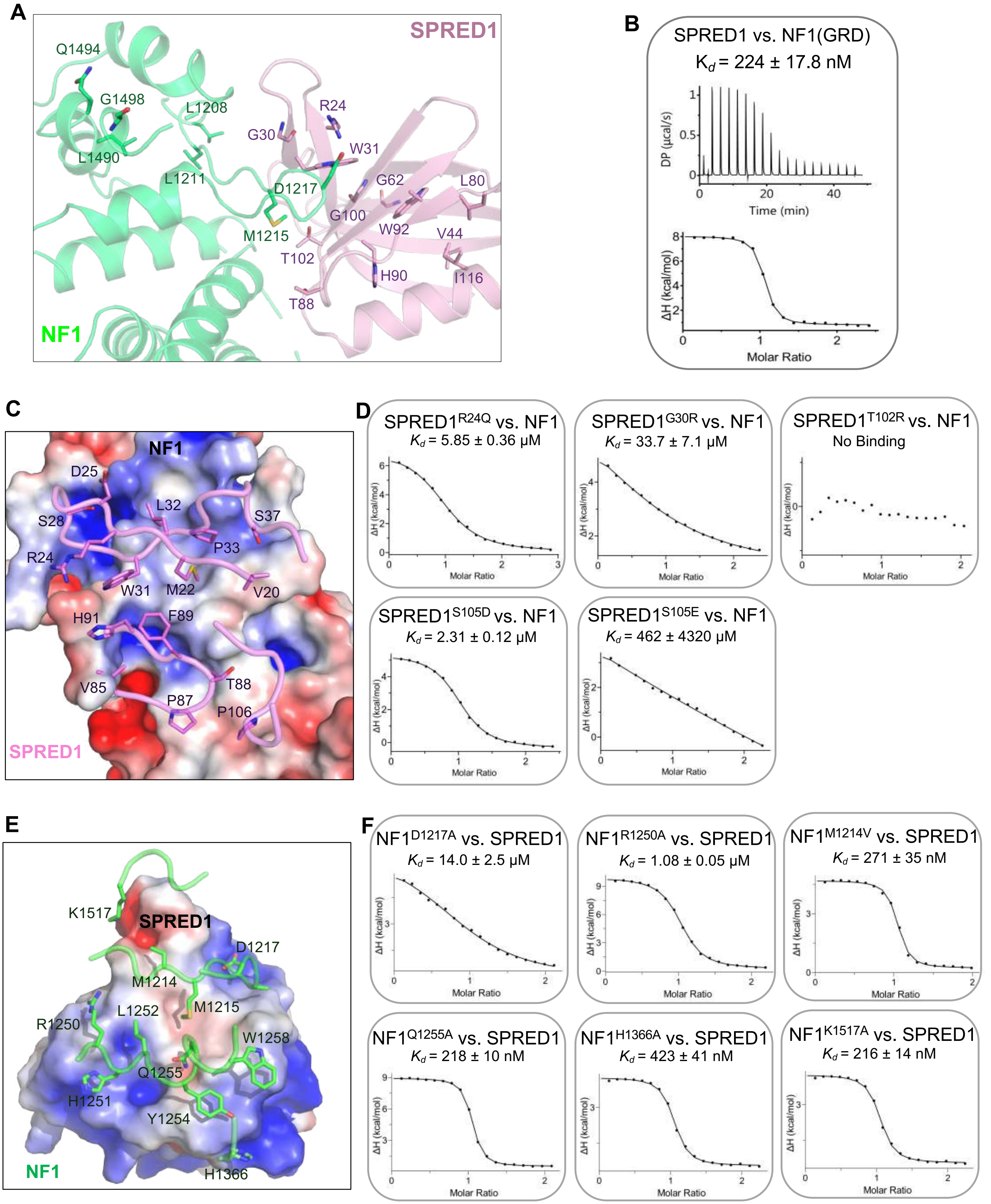

Figure S3. The SPRED1(EVH1)-NF1(GRD) interaction interface and the impact of SPRED1 and NF1 mutations observed in Legius syndrome and neurofibromatosis type 1 disease. (**A**) Mapping the pathogenic mutations observed

in SPRED1 (light pink) and NF1 (green) on the SPRED1-NF1 complex present in the ternary complex. (**B**) ITC titration experiment to measure the dissociation constant between NF1(GRD) and SPRED1(EVH1). (**C**) SPRED1(EVH1)-NF1(GRD) interaction interface, with NF1 shown in electrostatic surface representation and the SPRED1 regions that participate at the interface shown in cartoon mode and colored light pink. The SPRED1 residues that are important for the interaction are highlighted in stick representation. (**D**) Dissociation constant of SPRED1 mutants with NF1(GRD) measured using ITC titration experiments. (**E**) SPRED1(EVH1)-NF1(GRD) interaction interface, with SPRED1 shown in electrostatic surface representation and the NF1 regions that participate at the interface shown in cartoon mode and colored green. The NF1 residues that are important for the interaction are highlighted in stick representation. (**F**) Dissociation constant of NF1(GRD) mutants with SPRED1(EVH1) measured using ITC titration experiments.

Related to Figure 4.

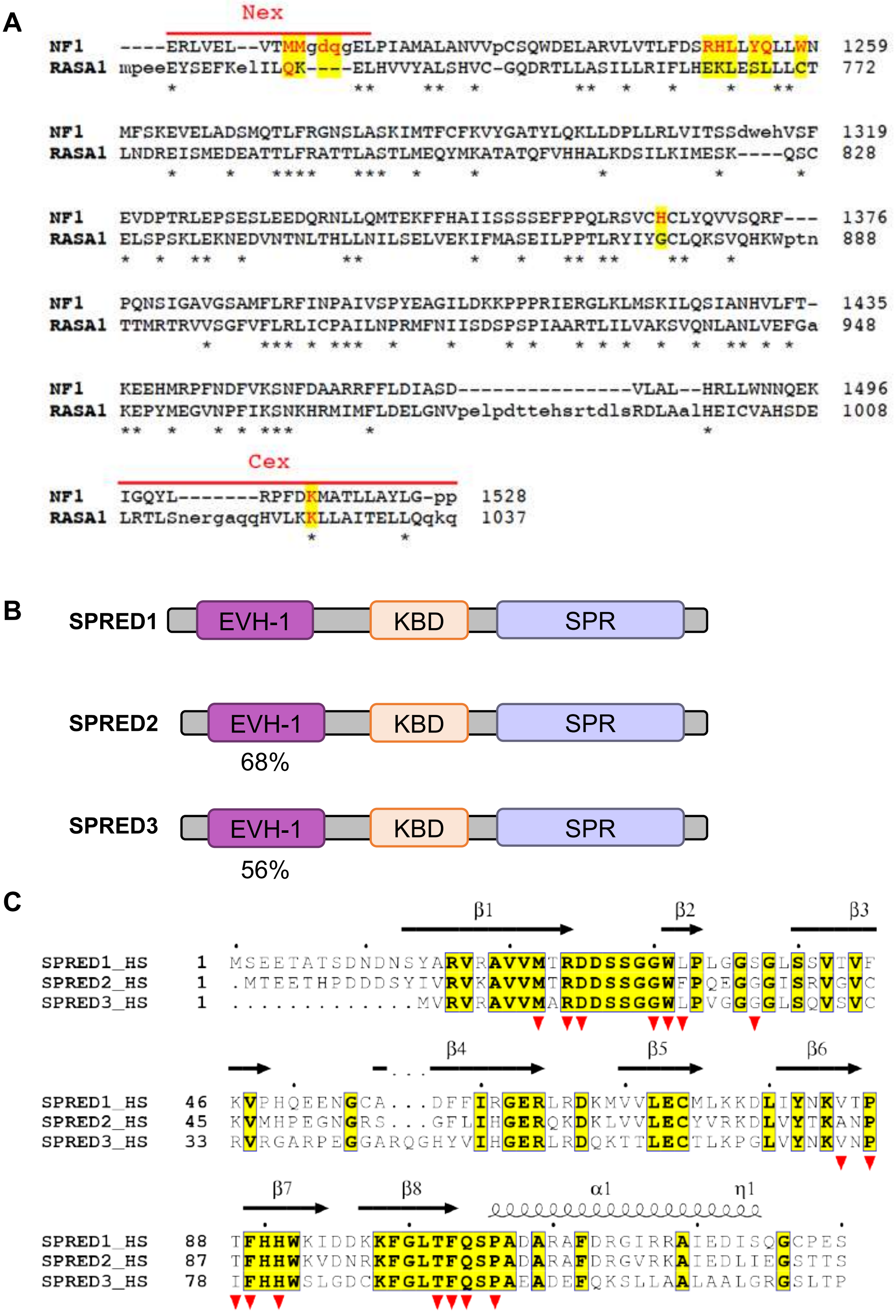

Figure S4. Sequence analysis of human RasGAP and SPRED proteins.
(**A**) Amino acid sequence alignment between NF1(GRD) and RASA1(GRD). The conserved residues between RASA1 and NF1 are marked with a star. The NF1 residues that participate in the interaction with SPRED1 are highlighted in yellow. The N- and C-terminal residues that form the GAPex region are shown in the alignment (red line). (**B**) Domain architecture of human SPRED1, SPRED2, and SPRED3.

The three conserved domains, EVH1, KBD, and SPR, are shown in different colors. The sequence identity of residues located in the EVH1 domain of SPRED1, SPRED2, and SPRED3 are shown under the panels (compared to SPRED1). (**C**) Amino acid sequence alignment of human SPRED1, SPRED2, and SPRED3. Conserved residues among the SPRED family are highlighted in yellow. The secondary structure of SPRED1 is shown above the alignment. The SPRED1 residues that are involved in the interaction with NF1(GRD) are shown with red inverted triangles.

Related to Figure 5.

A

B

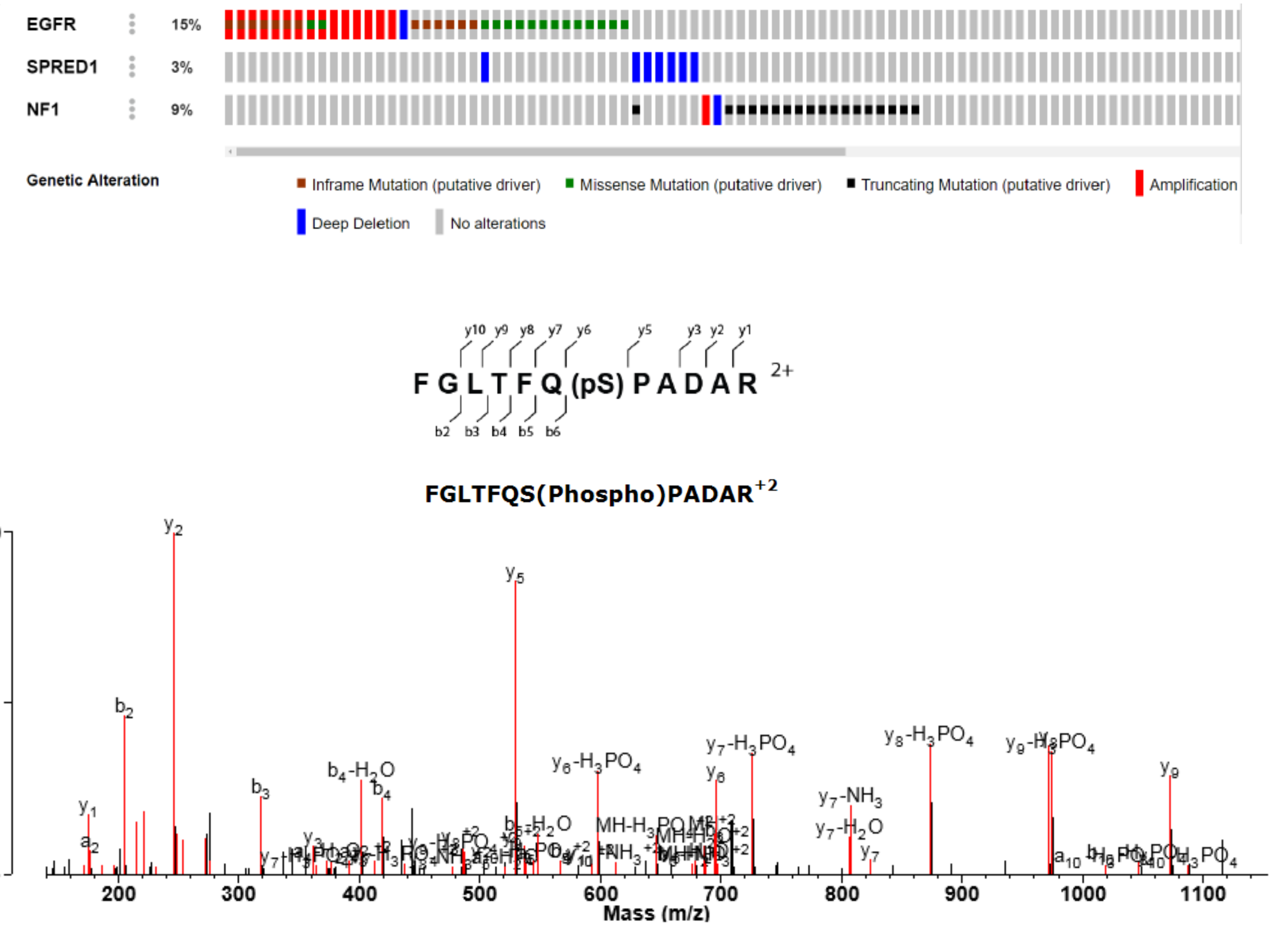

Figure S5. Lung adenocarcinoma genetics and phospho-SPRED1(S105) mass spectrometry. (**A**) EGFR, SPRED1, and NF1 human lung adenocarcinoma genetics with visualization using cBioPortal (n=230). Variants of unknown significance were excluded. (**B**) Spectrum and annotated phospho-SPRED1(S105) peptide.

Related to Figure 6.

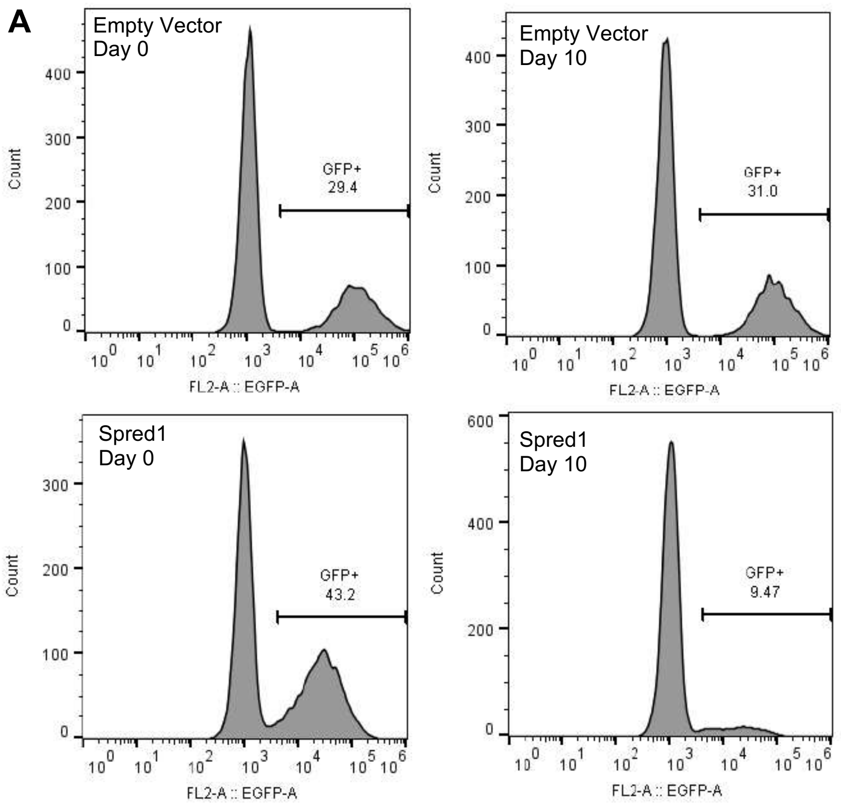

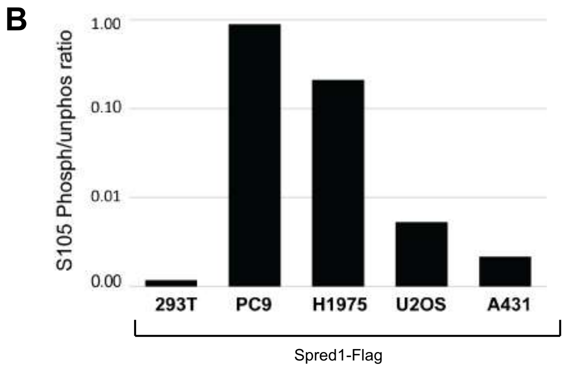

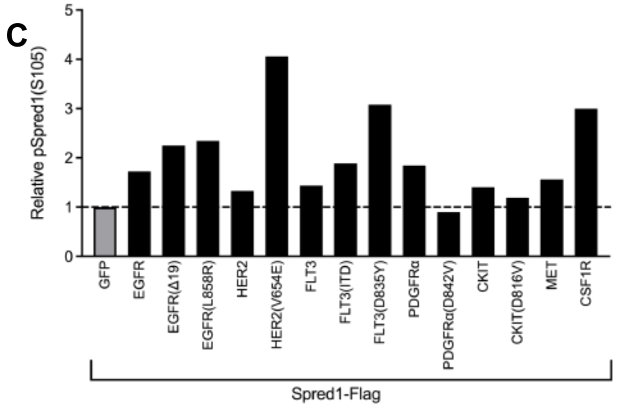

D

pSite	GFP	EGFR	EGFR(Δ19)	EGFR(L858R)	HER2	HER2(V654E)	FLT3	FLT3(ITD)	FLT3(D835Y)	PDGFRα	PDGFRα(D842V)	CKIT	CKIT(D816V)	MET	CSF1R
pS38	1.00	1.07	0.78	0.83	0.52	0.54	0.96	0.64	0.71	0.93	1.11	1.26	2.40	1.27	3.14
pS105	1.00	1.72	2.25	2.34	1.33	4.06	1.44	1.89	3.08	1.84	0.90	1.40	1.19	1.56	3.00
pS147	1.00	2.82	9.09	8.60	2.38	3.61	2.64	1.19	1.52	0.74	0.81	0.59	9.03	1.30	0.04
pY168	1.00	5.72	0.69	3.72	4.04	7.10	19.91	7.84	15.52	2.06	39.03	0.16	143.21	0.67	3.13
pY187	1.00	1.62	0.25	1.47	1.77	1.36	7.30	3.07	6.50	3.07	15.10	0.78	71.69	0.60	6.47
pS238	1.00	1.07	0.69	1.06	0.64	0.93	1.51	1.08	1.36	1.00	1.32	0.88	1.54	1.00	0.97
pY292	1.00	44.44	22.99	57.45	57.24	68.66	54.37	22.43	40.86	16.87	109.80	1.30	332.89	4.92	6.70
pY294	1.00	290.16	119.17	490.90	328.34	472.33	427.93	108.56	298.78	48.18	279.01	9.19	2581.67	53.88	0.00

Figure S6: Representative flow cytometry GFP histograms and RTK panel screen. (**A**) Representative K562 flow cytometry histograms from SPRED1-IRES-GFP and Empty Vector-IRES-GFP-expressing retrovirus cells. The competition experiment began 3 days after infection, indicated by day 0, and concluded on day 10. (**B**) Proportion of phosphorylated to unphosphorylated SPRED1 at the S105 site was estimated as a ratio of intensities of phosphorylated and unphosphorylated S105-containing peptides detected by MS. (**C**) RTK panel screen for phosphorylated SPRED1(S105). (**D**) Heat map of all SPRED1 phosphorylation sites with RTK expression from LC-MS/MS.

Related to Figure 7.

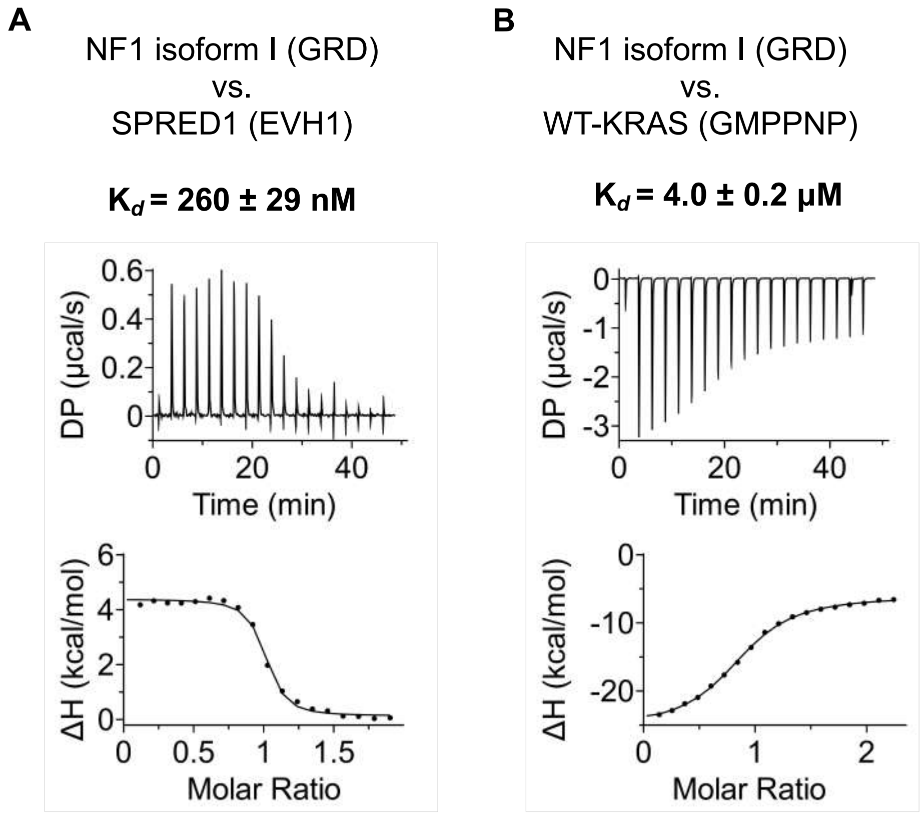

Figure S7. Effect of 23 amino acid insertion in the GAP-related domain of NF1 isoform I on its ability to interact with SPRED1(EVH1) and KRAS-GMPPNP (**A-B**) ITC titration experiment to measure the dissociation constant between (A) SPRED1(EVH1) and NF1 isoform I(GRD), (B) KRAS(WT)-GMPPNP and NF1 isoform I (GRD).

Related to Figure 1.

Table S1: Crystallographic data collection and refinement statistics.

	WT-KRAS(GMPPNP)-NF1(GRD)-SPRED1(EVH1)	Q61L-KRAS(GMPPNP)-NF1(GRD)-SPRED1(EVH1)
**Data collection**		
Resolution (Å)	79.18 – 2.76 (2.92 – 2.76) *	78.33 - 2.54 (2.69 - 2.54)
Space group	P 1 2_1 1	P 1 2_1 1
Cell dimensions		
a, b, c (Å)	72.6, 70.6, 80.3	72.0, 70.2, 79.7
α, β, γ (°)	90.0, 99.6, 90.0	90.0, 100.7, 90.0
Mean I/sigma(I)	11.7 (2.0)	11.5 (1.6)
Completeness (%)	97.6 (95.2)	98.2 (95.0)
Redundancy	4.3 (4.3)	3.4 (3.3)
Wilson B Factor (Å^2)	65.53	53.50
R-meas	0.083 (0.709)	0.082 (0.954)
R-merge	0.073 (0.624)	0.069 (0.802)
CC1/2	0.998 (0.873)	0.998 (0.801)
**Refinement**		
No. reflections	20174	25425
R_{work}/ R_{free}	0.2208/0.2449	0.2160/0.2639
Number of non-H atoms	4606	4636
Protein	4555	4565
Ligand	46	46
Water	5	25
B-factors (Å^2)	96.3	87.9
Protein	96.5	88.2
Ligand	71.4	66.4
Water	80.6	70.6
Ramachandran plot		
Favored	97.53%	96.30%
Allowed	2.47%	3.70%
R.m.s deviations		
Bond lengths (Å)	0.004	0.006
Bond angles (°)	0.66	0.80

*Highest resolution shell is shown in parenthesis.

Related to Figure 1.

Table S2: **A summary of residue-pairs that are involved in hydrogen bond, salt-bridge and hydrophobic interactions at the KRAS-NF1 interface in the structure of SPRED1-NF1-KRAS complex**.

	**Hydrogen Bond**		**Salt Bridge**		**Hydrophobic Interaction**	
**KRAS Region**	**KRAS**	**NF1**	**KRAS**	**NF1**	**KRAS**	**NF1**
**Switch I**	Y32 O	R1276 Nε	E37 Oε1	K1419 Nζ	Y32	R1276
	Y32 Oη	G1277 N	D38 Oδ1	K1423 Nζ	Y32	G1277
	D33 Oδ1	N1430 Nδ2			Y32	Q1272
	E37 O	K1423 Nζ			D33	N1430
	S39 Oγ	R1325 Nη2			P34	R1276
	S39 N	E1437 Oε1			P34	L1390
					T35	K1436
					I36	S1422
					E37	K1419
					D38	E1437
					D38	K1423
					D38	K1436
					S39	R1325
					Y40	K1436
					R41	R1435
**Switch II**	Q61 Nε2	G1277 O	E62 Oε1	K1283 Nζ	G60	N1278
	E62 N	N1278 Oδ1	E63 Oε1	K1283 Nζ	Q61	G1277
	E63 Oε1	T1286 Oγ1	E63 Oε2	R1391 Nε	Q61	R1391
	E63 Oε2	N1278 Nδ2			E62	N1278
	Y64 Oη	L1390 O			E62	K1283
					E63	R1391
					E63	P1395
					E63	N1278
					E63	T1286
					Y64	L1390
					Y64	P1395
					Y64	N1394
					A66	S1399
					A66	E1402
					M67	V1398
					Q70	K1419
**Other Regions**	Q25 Nε2	K1436 O	D54 Oδ1	R1325 Nη1	S17	K1436
			D54 Oδ2	R1325 Nη2	Q25	K1436
					D54	R1325
					K88	C1233

Related to Figure 2.

Table S3: **A summary of residue-pairs that are involved in hydrogen bond, salt-bridge and hydrophobic interactions at the SPRED1-NF1 interface in the structure of SPRED1-NF1-KRAS complex**.

	**Hydrogen Bond**		**Salt Bridge**		**Hydrophobic Interaction**	
**NF1 Region**	**NF1**	**SPRED1**	**NF1**	**SPRED1**	**NF1**	**SPRED1**
**N-ter GAPex**	M1215 N	W31 O	D1217 Oδ1	R24 Nη1	E1210	L32
	M1215 O	W31 N	D1217 Oδ2	R24 Nη1	M1214	S28
	D1217 Oδ1	W31 Nε			M1214	G30
					M1214	W31
					M1215	G30
					M1215	W31
					M1215	T102
					D1217	R24
					D1217	W31
					Q1218	V85
					Q1218	T86
					G1219	F89
**GAPmin**	Q1255 Oε1	T88 Oγ			L1252	T102
	Q1255 Nε2	T88 Oγ			Y1254	T88
					Y1254	P106
					Q1255	P87
					Q1255	T88
					Q1255	F89
					W1258	P87
					H1366	P106
					D1464	A107
**C-ter GAPex**	K1517 Nζ	S27 Oγ			K1517	S27
	K1517 Nζ	S28 Oγ			K1517	S28

Related to Figure 4.
